# *Burkholderia pseudomallei* in a lowland rice paddy: seasonal changes and influence of soil depth and physico-chemical properties

**DOI:** 10.1038/s41598-017-02946-z

**Published:** 2017-06-08

**Authors:** L. Manivanh, A. Pierret, S. Rattanavong, O. Kounnavongsa, Y. Buisson, I. Elliott, J. -L. Maeght, K. Xayyathip, J. Silisouk, M. Vongsouvath, R. Phetsouvanh, P. N. Newton, G. Lacombe, O. Ribolzi, E. Rochelle-Newall, D. A. B. Dance

**Affiliations:** 1 Institut de la Francophonie pour la Médecine Tropicale, BP 9519 Vientiane, Lao People’s Democratic Republic; 2 Lao-Oxford-Mahosot Hospital-Wellcome Trust Research Unit, Microbiology Laboratory, Mahosot Hospital, Vientiane, Lao People’s Democratic Republic; 3 iEES-Paris (IRD-Sorbonne Universités -UPMC-CNRS-INRA-UDD-UPEC), c/o Department of Agricultural Land Management (DALaM), Vientiane, Lao People’s Democratic Republic; 4 Centre for Tropical Medicine & Global Health, University of Oxford, Oxford, UK; 5 iEES-Paris (IRD-Sorbonne Universités -UPMC-CNRS-INRA-UDD-UPEC), c/o Soils and Fertilizers Research Institute (SFRI), Hanoi, Vietnam; 6 International Water Management Institute (IWMI), Southeast Asia Regional Office, PO BOX 4199 Vientiane, Lao People’s Democratic Republic; 7 Géosciences Environnement Toulouse – GET (IRD, Université de Toulouse, CNES, CNRS, UPS), 31400 Toulouse, France; 8 iEES-Paris (IRD-Sorbonne Universités -UPMC-CNRS-INRA-UDD-UPEC), Université Pierre et Marie-Curie, Paris, France; 9 Faculty of Infectious and Tropical Diseases, London School of Hygiene and Tropical Medicine, London, UK

## Abstract

Melioidosis, a severe infection with the environmental bacterium *Burkholderia pseudomallei*, is being recognised increasingly frequently. What determines its uneven distribution within endemic areas is poorly understood. We cultured soil from a rice field in Laos for *B. pseudomallei* at different depths on 4 occasions over a 13-month period. We also measured physical and chemical parameters in order to identify associated characteristics. Overall, 195 of 653 samples (29.7%) yielded *B. pseudomallei*. A higher prevalence of *B. pseudomallei* was found at soil depths greater than the 30 cm currently recommended for *B. pseudomallei* environmental sampling. *B. pseudomallei* was associated with a high soil water content and low total nitrogen, carbon and organic matter content. Our results suggested that a sampling grid of 25 five metre square quadrats (i.e. 25 × 25 m) should be sufficient to detect *B. pseudomallei* at a given location if samples are taken at a soil depth of at least 60 cm. However, culture of *B. pseudomallei* in environmental samples is difficult and liable to variation. Future studies should both rely on molecular approaches and address the micro-heterogeneity of soil when investigating physico-chemical associations with the presence of *B. pseudomallei*.

## Introduction

*Burkholderia pseudomallei*, the causative agent of the disease melioidosis, is an environmental bacterium that is widespread in soil and water in endemic areas, most notably Southeast Asia and northern Australia^1, 2^. With an estimated global burden of 165,000 human melioidosis cases per year, from which ~89,000 people die, melioidosis is probably endemic in at least 79 countries^3^. In Laos, infection is most frequently seen in rice farmers and is thought to be acquired mainly by inoculation whilst working in rice paddy, although inhalation of aerosolised bacteria, and aspiration and possibly ingestion of contaminated water are other potential modes of acquisition^4, 5^. The incidence of melioidosis varies across endemic areas and this is thought, at least in part, to relate to the uneven distribution of *B. pseudomallei* in the environment, which varies on both a large (e.g. regional/national) and a small (e.g. within a single field) scale^6–8^. The factors that determine the ability of a given environment to sustain the persistence or proliferation of *B. pseudomallei* are poorly understood. On a large scale, water content, temperature, pH, salinity, iron content, rainfall, other soil flora, vegetation, soil disturbance, the presence of animals, and soil type have all been suggested to have an influence on the distribution of *B. pseudomallei*, although the results of different studies have varied widely^2, 9–20^. Less is known about the factors that determine its distribution at a small scale, such as within a single rice paddy. It has recently been recommended that studies of *B. pseudomallei* in soil should routinely collect samples at 30 cm depth^21^. It has, however, also been suggested that the organism persists in the deeper clay layers of soil during the dry season, rising to the surface with the water table during the rainy season^22^. In this study we investigated the seasonal changes in the presence of *B. pseudomallei* at different depths (from near the surface to 90 cm below the surface) and places within the same rain-fed lowland rice field and attempted to correlate these with a range of physicochemical parameters in an effort to identify factors that may account for the small-scale variation in the distribution of *B. pseudomallei* within the environment.

## Results

### Detection of *B. pseudomallei*

The results of the culture of soil samples for *B. pseudomallei* are shown in Table 1 and Supplementary Table S1. The number of samples collected in each sampling round varied, as it was often impossible to collect the deeper samples during the rainy season due to the holes filling with water. Overall, 195 of 653 samples (29.7%) yielded *B. pseudomallei* and 113 of 653 samples (17.3%) yielded *B. thailandensis*. Eight samples, all collected during the rainy season, contained both species. Since the focus of the study was *B. pseudomallei* and the method was not specifically designed to detect *B. thailandensis*, further analysis of the latter was not undertaken. The quantitative estimates of the number of *B. pseudomallei* ranged from the limit of detection (i.e. detected on enrichment culture only) to 8,150 CFU g^−1^. However, it was often extremely difficult to count the numbers of *B. pseudomallei* colonies as a consequence of overgrowth by large numbers of competing flora. Furthermore, there were often significant discrepancies between the quantitative *B. pseudomallei* counts estimated from different plates, and the enrichment culture was negative for 88 of 195 culture-positive samples (45.1%).

**Table 1:** Isolation of *B. pseudomallei* at different soil depths for each sampling round.

Sampling Depth (cm)	April 2011	June 2011	Nov. 2011	April 2012
5 to 90	64/196 (32.7)	22/116 (19.0)	46/149 (30.9)	63/192 (32.8)
5	3/49 (6.1)	6/49 (12.2)	8/49 (16.3)	6/49 (12.2)
30	14/49 (28.6)	12/49 (24.5)	16/49 (32.7)	13/49 (26.5)
60	20/49 (40.8)	4/18 (22.2)	22/49 (45)	22/49 (44.9)
90	27/49 (55.1)	0/0	0/2 (0)	22/45 (48.9)

(Positive samples/Total collected and percentage in parentheses).

#### Seasonal variation

Of the 196 samples collected at the end of the dry season in April 2011, 32.7% (64 samples) contained *B. pseudomallei* (Table 1). The proportion of positive samples dropped to 19.0% (22 out of 116 samples) after the first monsoon rains in June 2011 but rose again to 30.9% (46 out of 149 samples) in November 2011 and 32.8% (63 out of 192 samples) in April 2012.

#### Association with depth

The probability of finding *B. pseudomallei* positive at 5 cm never exceeded 16% (November 2011) while it varied between 25 to 32% at 30 cm and exceeded 40% at soil depths of 60 and 90 cm (when all 49 quadrats could be sampled, i.e. only in the late dry seasons; Table 1). Towards the end of both the dry and rainy seasons, the proportion of positive samples significantly increased with depth. In April 2011, November 2011 and April 2012, Spearman’s correlation coefficients between *B. pseudomallei* counts and soil depth were 0.39 (p = 9.8 × 10^–9^, n = 196), 0.27 (p = 0.001, n = 149) and 0.34 (p = 1.75 × 10^−6^, n = 192), respectively (Supplementary Figure S1). Similarly, positivity (defined here as the number of times a quadrat yielded positive CFU counts) was positively correlated with soil depth (Spearman’s **ρ** = 0.27, p = 0.0001, n = 196). Although there were more positive samples closer to the soil surface in June and November 2011 than in April 2011 and 2012, this increase did not correspond to a detectable significant trend (P > 0.05).

### Dynamics of *B. pseudomallei* distribution and spatial auto-correlation

Mapping of log *B. pseudomallei* counts revealed variations in the bacterial count with time, depth and space. The highest point-wise densities of *B. pseudomallei*, of 8,150, 6,778, 4,050, 3,980 and 3,100 CFU g^−1^, were found in April 2012 at 60 cm, November 2011 at 60 cm, April 2012 at 90 cm, April 2011 at 90 cm and June 2011 at 30 cm, respectively. The lowest point-wise densities of *B. pseudomallei* (<1,000 CFU g^−1^) were consistently found at soil depths of 5 and 30 cm at all sampling rounds, except for June 2011.

There was a significant (p < 0.001) trend of mean log of CFU along the N-S direction in April 2011 at 60 cm and along the E-W direction in April 2012 at 30 cm (p < 0.05); in November 2011, at 30 cm, both mean and variance displayed a trend along the E-W direction (p < 0.05). Besides these three exceptions, all other distributions of *B. pseudomallei* counts did not apparently change with time. None of the 5 cm distributions were spatially auto-correlated (Table 2). Likewise, the April 2011 and 2012 distributions at 30 cm did not appear spatially auto-correlated. June and November 2011 distributions of *B. pseudomallei* counts at 30 cm appeared to correspond to long-range spatial trends. Only very few models of the empirical omnidirectional variograms yielded ranges less than the size of the measured plot, namely that corresponding to *B. pseudomallei* count distribution at 90 cm in April at 60 cm in November 2011 (ranges and nugget/sill ratios of 5.99 m and 0.15; 17.31 m and 0.13, respectively; Table 2). Directional variograms revealed some degree of anisotropy in the case of the *B. pseudomallei* count distributions of April 2011 at 60 and 90 cm (range = 13.16 and 13.93 m, respectively), and November 2011 at 60 cm (range = 14.01 m) along the E-W direction (Table 2). Overall, quadrats with high (low) *B. pseudomallei* count were only likely to sit next to quadrats with high (low) counts – i.e. be spatially auto-correlated - in a very limited number of the deepest observed distributions (Supplementary Figure S2).

**Table 2:** Summary of parameters obtained from fitting maximum likelihood (ml) and ordinary least squares (ols) models to omnidirectional (Omni) and directional (0 (N-S); 90 (E-W)) empirical semivariograms corresponding to the spatial distribution of log-transformed *B. pseudomallei* counts.

Date	Depth (cm)	Model	Direction	Range (m)	Nugget	Partial sill	Nugget/Sill
Apr-11	5	ml	Omni	0.29	0.28	0.11	0.72
		ols	Omni	12.91	0.4	0	1
		ols	0 (N-S)	89.74	0.35	0	1
		ols	90 (E-W)	78.71	0.44	0	1
Apr-11	30	ml	Omni	0.29	1.81	0.66	0.73
		ols	Omni	9.85	2.51	0	1
		ols	0 (N-S)	158.33	2.5	0	1
		ols	90 (E-W)	150.12	2.53	0	1
Apr-11	60	ml	Omni	14.28	3.35	1.34	0.71
		ols	Omni	76601.55	3.52	4650.49	0
		ols	0 (N-S)	118853.07	3.04	6042.47	0
		***ols***	***90 (E-W)***	***13.16***	***2.48***	***3.75***	***0.4***
Apr-11	90	ml	Omni	5.15	1.47	4.12	0.26
		***ols***	***Omni***	***5.99***	***0.96***	***5.28***	***0.15***
		ols	0 (N-S)	199.61	4.55	0	1
		***ols***	***90 (E-W)***	***13.93***	***1.18***	***7.96***	***0.13***
Jun-11	5	ml	Omni	0.29	2.1	0.77	0.73
		ols	Omni	9.71	2.93	0	1
		ols	0 (N-S)	93.34	3.07	0	1
		ols	90 (E-W)	80.91	2.83	0	1
Jun-11	30	ml	Omni	0.31	3.27	1.34	0.71
		ols	Omni	173049.15	3.5	11381.57	0
		ols	0 (N-S)	171597.91	3.23	16229.19	0
		ols	90 (E-W)	180796.27	3.83	6532.55	0
Nov-11	5	ml	Omni	0	1.38	0	1
		ols	Omni	9.94	1.41	0	1
		ols	0 (N-S)	90.49	1.33	0.76	0.64
		ols	90 (E-W)	79.59	1.35	0	1
Nov-11	30	ml	Omni	4.88	1.5	0.45	0.77
		ols	Omni	87855.91	1.43	2554.33	0
		ols	0 (N-S)	157.7	1.75	0	1
		ols	90 (E-W)	65914.29	1.13	3689.2	0
Nov-11	60	ml	Omni	6.92	3.67	2.91	0.56
		***ols***	***Omni***	***17.31***	***3.55***	***5.12***	***0.41***
		ols	0 (N-S)	157.75	4.72	6.38	0.42
		***ols***	***90 (E-W)***	***14.01***	***2.04***	***8.26***	***0.2***
Apr-12	5	ml	Omni	0.32	0.75	0.31	0.71
		ols	Omni	198.54	0.83	2.88	0.22
		ols	0 (N-S)	110679.06	0	9357.37	0
		ols	90 (E-W)	41.46	0.71	0	1
Apr-12	30	ml	Omni	0.86	0	4.36	0
		ols	Omni	125187.43	3.96	3230.86	0
		ols	0 (N-S)	175020.39	3.93	7019.37	0
		ols	90 (E-W)	73569.51	3.93	1360.14	0
Apr-12	60	ml	Omni	0.98	0	7.33	0
		ols	Omni	155021.53	6.48	8358.49	0
		ols	0 (N-S)	77704.54	6.24	3869.43	0
		ols	90 (E-W)	232340.58	6.57	14583.43	0
Apr-12	90	ml	Omni	0	8.84	0	1
		ols	Omni	5.98	9.03	0	1
		ols	0 (N-S)	8.71	9.43	0	1
		ols	90 (E-W)	4.01	7.41	1.41	0.84

Italicized bold lines correspond to models from which spatial auto-correlation could be inferred.

### Quantitation of *B. pseudomallei*

**Figure 1:**
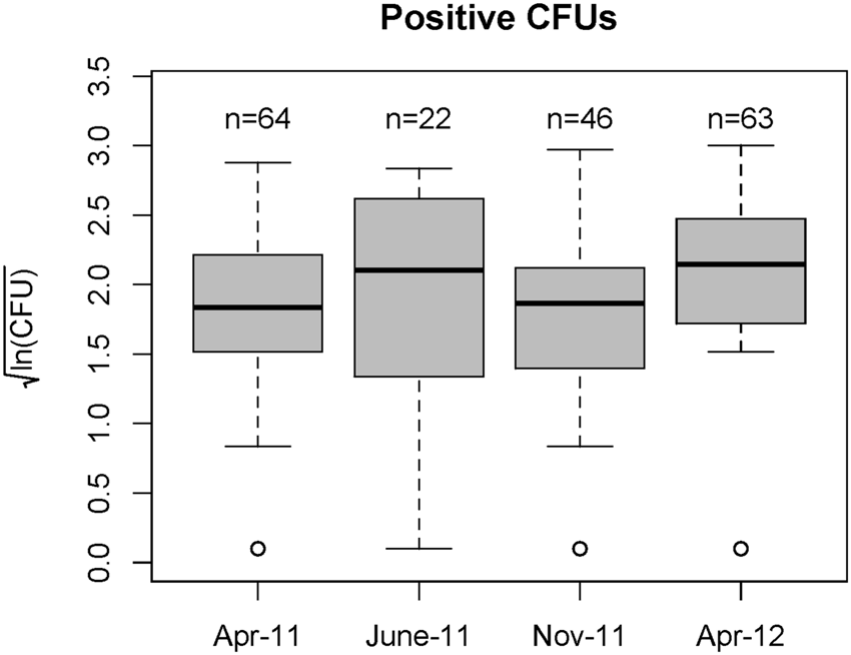
Quantitation of *B. pseudomallei*. Box-whisker plots log-transformed *B. pseudomallei* counts for each sampling round. The central horizontal line indicates the median value, and the upper and lower edges of boxes (hinges) correspond to the 25th and 75th percentile values, while the whiskers extend 1.5× beyond the spread of the hinges. Data points outside this range (outliers) are indicated with circles.

When all depths were aggregated for each sampling round, the skewness, median and mean of counts of *B. pseudomallei* of positive samples were 6.39, 29 (169) CFU g^−1^, 1.50, 97 (584) CFU g^−1^, 4.45, 32 (321) CFU g^−1^, and 8.09, 100 (523) CFU g^−1^ in April 2011, June 2011, November 2011 and April 2012, respectively (Fig. 1). Overall, there were no differences between the means of *B. pseudomallei* counts corresponding to the four sampling rounds.

#### Associations between soil properties and *B. pseudomallei* counts

**Figure 2:**
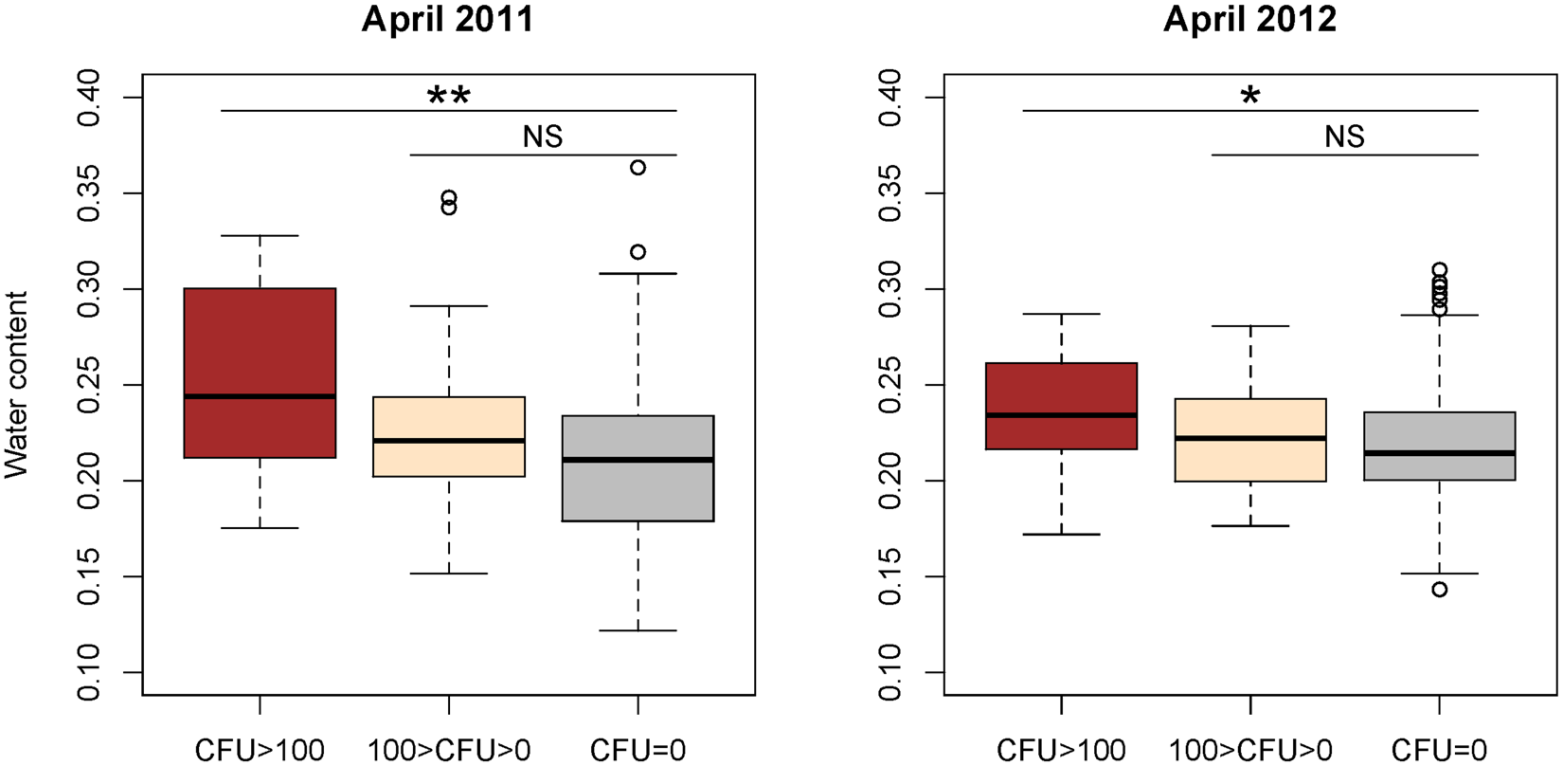
Box-whisker plots of soil water content of samples in which *B. pseudomallei* was abundant (CFU > 100), present (100 > CFU > 0), or absent (CFU = 0) in April 2011 and April 2012. Refer to Fig. 1 for the explanation of Box–whisker plots. *P ≤ 0.05, **P ≤ 0.005 and NS = Not Significant.

Of the soil properties measured, only soil water content, total nitrogen, total carbon and organic matter content significantly differed between *B. pseudomallei-*positive and -negative samples (henceforward referred to simply as positive and negative samples only). A Kruskal-Wallis test revealed that in the late dry seasons (April 2011 and April 2012), the soil water content was significantly associated with the *B. pseudomallei* CFU g^−1^ (**χ** ^2^_(2)_ = 13.581, p < 0.005 and **χ** ^2^_(2)_ = 6.439, p < 0.05, in April 2011 and April 2012, respectively). A post-hoc test using Mann-Whitney tests with Bonferroni correction suggested that the water content of samples with *B. pseudomallei* present at >100 CFU g^−1^ was higher than that of negative samples (p < 0.005, r = 0.166 and p < 0.05, r = 0.129, in April 2011 and April 2012, respectively) but not different from that of samples with *B. pseudomallei* present at only 0–100 CFU g^−1^ (Fig. 2). In other words, in the late dry seasons, *B. pseudomallei* was more abundant when soil moisture was high, which is consistent with its prevalence in deeper soil horizons.

When pooling all the data from the four sampling rounds together, it also appears that the highest CFU values (counts ≥ 100 CFU g^−1^) occur in soil that is significantly more moist (Water Content = 0.243 (n = 56) vs. 0.228 (n = 204), p < 0.05; Welch Two Sample t-test) than soil from in which negative and lower CFU counts were found.

Nitrogen, organic carbon and organic matter contents were significantly lower for *B. pseudomallei* positive than negative samples in April 2011 and April 2012 but not in June and November 2011 (Table 3).

**Table 3:** Nitrogen (N), organic carbon (Org. C) and organic matter (O.M.) contents of *B. pseudomallei* positive and negative samples; U: Mann-Whitney’s U test; n: sample size; significance level: NS: non significant; *p < 0.05, **p < 0.01.

	N (g kg^−1^)			Org. C (g kg^−1^)			O.M. (g kg^−1^)		
	Positive	Negative	U	Positive	Negative	U	Positive	Negative	U
*April 2011*	0.29	0.47	260^*^	2.37	4.56	260.5^*^	4.11	7.90	261^*^
*n*	25	33		25	33		25	33	
*June 2011*	0.38	0.56	53^NS^	3.14	5.56	47^NS^	5.44	9.62	47^NS^
*n*	6	25		6	25		6	25	
*Nov. 2011*	0.33	0.53	106^NS^	2.80	5.28	104.5^NS^	4.85	9.13	105^NS^
*n*	13	26		13	26		13	26	
*April 2012*	0.30	0.51	238^**^	2.60	5.03	242.5^*^	4.5	8.70	243^*^
*n*	32	25		32	25		32	25	

## Discussion

Melioidosis is being increasingly recognised as a significant public health problem both worldwide^3^ and in Laos since the organism was first isolated from soil and the first human cases were described just over a decade ago^23, 24^. *B. pseudomallei* has been recognised as having an environmental reservoir since French workers succeeded in isolating it from soil and water in the middle of the last century^25, 26^. Despite this, and despite the major advances that have been made in our understanding of the pathogen in recent years, the ecology and distribution of *B. pseudomallei* are still poorly understood. Soil is an extraordinarily complex ecosystem, and there is a remarkable diversity of *B. pseudomallei* concentrations and genotypes within soil in melioidosis-endemic areas^6, 8, 27^. It is likely that a range of interacting climatic, physico-chemical and biological factors are involved in determining whether *B. pseudomallei* will survive and proliferate in a given location. A better understanding of these factors would help us generate risk maps for the occurrence of melioidosis and might possibly lead to the development of measures to reduce or remove the organism from already colonised environments. We therefore chose to carry out this study over a period of 13 months, at the scale of a paddy field, in a region of Laos highly endemic for melioidosis.

*B. pseudomallei* was present in our four sampling cycles with relatively constant isolation rates, except after the early season rains, which may be due to the fact that some of the deeper samples could not be collected at this time due to waterlogging. The impossibility of collecting samples at greater depths when the field was flooded, which is arguably the time when the exposure of rice farmers to *B. pseudomallei* is maximum, was one of the limitations of this study. Similar studies to detect *B. pseudomallei* in the environment by culture at different times of the year have given discordant results. While melioidosis undergoes seasonal increases in incidence during the rainy season, several studies have found paradoxically that there was a higher isolation rate of *B. pseudomallei* from the environment during the dry season^28, 29^ whereas Kaestli *et al*. found that this was true for residential properties but not for undisturbed sites^12^. In contrast, some studies have found higher isolation rates of *B. pseudomallei* from soil in the rainy season^20^.

Our key findings were that the organism was detectable in some samples at all levels throughout the year, more abundantly at 60 and 90 cm than at 5 and 30 cm. This corroborates the concept that the organism can persist in the deeper layers of soil during the dry season and that it rises to the surface during the rainy season^22, 28^. There are discrepancies between the various studies aimed at comparing the yields of *B. pseudomallei* culture at different depths in the soil. Thus, Wuthiekanun *et al*. found the highest isolation rates at the deepest levels they sampled, 60 cm during the wet season and 90 cm during the dry season^28^. Other studies have also found lower positivity rates near the soil surface than at deeper levels^22, 28–30^, whereas Palasatien *et al*. found higher positivity rates at 15 and 30 cm than 45 cm depth^31^. That the overall yield in our study was higher at depth than near the surface means that the recent recommendation to standardise sampling depth at 30 cm based on studies conducted in Thailand^21^, whilst pragmatic, could result in other areas with lower level *B. pseudomallei* contamination being falsely labelled negative. At our study site, collecting samples at soil depths of at least 60 cm increased the probability of sampling points being positive *for B. pseudomallei* from about 30% to over 40%. This difference might possibly relate to different maximum depths of the water table. Given this higher probability of finding positive samples at greater depth, however, we conclude that, in environments such as Asian paddy fields, only 10 independent samples randomly taken in one area at a depth of at least 60 cm is sufficient to detect *B. pseudomallei* with confidence, as the 95% binomial confidence interval for 10 samples ranges from 0 to 30.8%^6^, which does not include a true probability of 40% of more. Further, given the range of spatial auto-correlation for CFU counts in the investigated plot (13 to 17 m in the E-W direction, when it could be determined), it appears that the quadrat size of 5 m squares used in this work is sufficient to capture the spatial variability of *B. pseudomallei* CFU counts, as the optimal distance between sampling points is considered to be half the range of the spatial auto-correlation observed in the semivariogram^32^. Further, while we did not attempt to sample at levels deeper than 90 cm, it remains plausible that *B. pseudomallei* is actually present below this depth. A study of microbial diversity in a paleosol collected 188 m below the soil surface showed that *Burkholderia* spp. were amongst the most frequent types of microorganisms identified in such a deep environment^33^. The presence of *B. pseudomallei* in 33% of bore water samples in northern Australia also supports such a possibility^34^. If true, this makes it extremely unlikely that it will be possible completely to eliminate *B. pseudomallei* from contaminated environments.

The soil in the field we studied was both relatively acidic (mean pH 4.31, range 3.38–5.58, Supplementary Table 3), and sandy (mean sand content 37%, range 12–84%, data not shown), as opposed to the clay soil traditionally associated with *B. pseudomallei* in Australia^11, 22, 35^, but similar to the melioidosis endemic area of north east Thailand studied by Palasatien^32^, located about 270 km south and on the other side of the Mekong river. Acid tolerance is a characteristic of the genus Burkholderia that is thought to give them a competitive advantage in acidic soils^36^. Palasatien and colleagues found that in north east Thailand the presence of *B. pseudomallei* was associated with sandy soil with a pH from 5.0–6.0, a moisture content >10%, and higher chemical oxygen demand and total nitrogen than negative sites, soil pH being the major determinant^31^. Acidic soil and water have been repeatedly associated with the presence of *B. pseudomallei* ^9, 10, 12, 29, 31, 37–40^. In northern Australia, Kaestli and colleagues found that the factors associated with the presence of *B. pseudomallei* differed between undisturbed sites and environmentally manipulated areas (moist areas rich in grasses or the presence of livestock animals, lower soil pH and different combinations of soil texture and colour respectively)^12^. Suebasri *et al*. reported that a higher soil pH 6.05, a low water holding capacity, and low iron were associated with the presence of *B. pseudomallei* in soil from paddy fields in north east Thailand in the rainy season, whereas a high concentration of manganese correlated with the presence of the organism in the dry season^20^. More recently, workers in both Thailand and Australia have reported an association between sandy, nutrient-depleted soil and the presence of *B. pseudomallei* ^14, 15^, contrasting with earlier reports of an association with soils enriched with organic matter from animal waste^12^.

**Figure 3:**
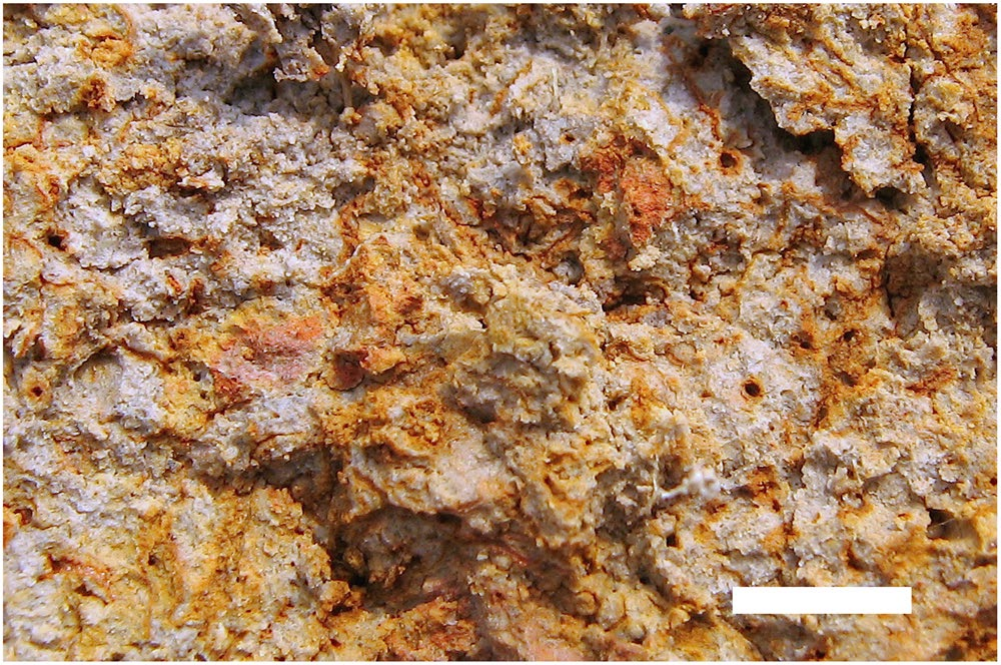
Close-up photograph of the typical soil structure in the subsoil horizons of the Ban Nabone site showing oxidized micro-domains (mostly along cracks and around root channels) in brown-red colours immediately adjacent to reduced micro-domains where greyish colours prevail. The white horizontal bar to the bottom right of the photograph represents 5 mm.

In our study, the occurrence of consistently positive samples at the same locations throughout the year suggests the possibility of spatial structuration of the distribution of *B. pseudomallei*. The most consistent finding was that, at all sampling rounds, positive samples tended to be located in wetter than average areas, albeit not necessarily in the wettest parts of the field. Soil water content is also one of the factors that has repeatedly been associated with the presence of *B. pseudomallei* ^10, 29–31^, although a recent study based on the analysis of 6,100 soil samples collected from 61 rice fields in Thailand found *B. pseudomallei* to be negatively associated with soil moisture^15^. In addition, in our study, samples in which *B. pseudomallei* was detected tended to have lower levels of carbon, nitrogen and organic matter, although this may be confounded by the higher positivity rate at greater depths. Hantrakun *et al*. also found *B. pseudomallei* to be negatively associated with soil organic matter content, concluding that *B. pseudomallei* may thrive in nutrient-depleted soils^15^. The data regarding iron are conflicting – the organism has been associated with both low^14, 15, 20^ and high^9, 16, 17^ levels of iron in natural environments, whilst iron enhanced the growth of *B. pseudomallei* in soil microcosms under laboratory conditions^35, 41^. *B. pseudomallei* is known to produce a siderophore and has other mechanisms that enable it to acquire iron. This and its nutritional versatility might conceivably give it a survival advantage in iron-depleted environments^42, 43^. This might also result in a less diverse flora in such places, making the selective isolation of *B. pseudomallei* relatively easier. However, the overall lack of consistency between reports attempting to establish correlations between the presence of *B. pseudomallei* and soil properties may actually be due to the use of large bulk soil samples (typically 100 cm^3^, as in this study) or composite soil samples. This is probably inappropriate as it likely mixes up a variety of physico-chemically contrasting micro-niches in which *B. pseudomallei* may or may not be present. Indeed, careful examination of soil structure at the site investigated in this study revealed the presence of redoximorphic features, consisting, at the sub-millimetre scale, of well aerated domains (mostly along cracks and around root channels), where oxidized or ferric (Fe^+3^) iron compounds are responsible for the brown, yellow and red colours visible in Fig. 3, next to more saturated domains from which iron reduced to the ferrous (Fe^+2^) form and greyish colours prevail. This heterogeneity of soil at a micro-scale seems to have been largely overlooked to date and may partly explain the contradictory nature of the literature on *B. pseudomallei* ecology. Investigating these micro-niches represents a potential avenue for advancing our understanding of the environmental determinants of *B. pseudomallei*.

One potential criticism of this, and many other studies of environmental *B. pseudomallei* ^6, 8, 15, 16, 20–23, 26–28, 31, 44–50^, is the use of culture to detect the organism. Whilst a positive culture is unequivocal evidence of the presence of the organism, negative cultures may be misleading. First, the organism may be present in a ‘viable non-cultivable’ state^11^. Secondly, it is extremely difficult to devise selective culture methods that will allow *B. pseudomallei* to grow whilst suppressing closely related environmental organisms that may have very similar nutritional requirements and susceptibility to inhibitors such as antibiotics. The challenge is very different from that of growing *B. pseudomallei* from clinical specimens, the purpose for which most selective media were originally developed. A wide range of methods has been used previously to detect the presence of *B. pseudomallei* in soil, including animal inoculation, culture in liquid or solid media, and molecular methods, particularly PCR. There have been several comparative studies of different culture methods that have yielded conflicting results. This topic has been recently extensively reviewed and consensus recommendations for culture methods published, albeit after we started this study^21^. During this study we used methods that had been employed successfully to isolate *B. pseudomallei* from soil sampled in Laos and elsewhere^47, 50^ and, having started this work using culture, we needed to use consistent methodology throughout the study to ensure comparability of results. It is possible that the yield would have been improved had we used a method employing polyethylene-glycol and sodium deoxycholate to liberate bacteria from the soil, that was published after this study had started^49^. However, we frequently observed culture plates with almost confluent growth of other flora in which it is extremely likely that *B. pseudomallei* could have been overlooked. Similar overgrowth of other organisms, potentially suppressing the growth of *B. pseudomallei*, is likely to have occurred in the liquid enrichment media, and may have accounted for the fact that enrichment cultures were frequently negative even when direct plating on solid media yielded *B. pseudomallei*. Remarkably, all quadrat/depth combinations but one yielded a negative culture at least once, suggesting that there were probably a number of false negative results. The enumeration of *B. pseudomallei* in soil samples using culture methods is also fraught with difficulty, and whilst we attempted this, as has been done in previous studies, we consider that the results should be treated with extreme caution.

It is thus likely that some of the confusing and conflicting data in the literature regarding *B. pseudomallei* ecology relates to shortcomings in the methods used. We are trying to develop improved culture methods to detect *B. pseudomallei* in soil, but increasingly believe that methods that are not based on culture, such as PCR^29^, are preferable for the study of environmental *B. pseudomallei*. PCR, with or without prior enrichment culture, has repeatedly been shown to give a higher yield of *B. pseudomallei* from environmental samples than culture alone^13, 29, 44, 45, 51, 52^. Based on the analysis of samples collected at the same rice paddy in 2013, we have also been able to confirm that the rate of detection from soil samples by qPCR following culture enrichment was significantly higher than that by culture methods^52^. Further work is still needed to define the optimal methods for the detection of environmental *B. pseudomallei*, but the use of appropriate methods is vital if we are to begin to unravel the complex interplay of factors that determine whether an environment will sustain the organism and thereby act as a source of melioidosis.

Despite clearly identified limitations, this study confirmed a higher prevalence of *B. pseudomallei* (from ~30% to >40%) at soil depths greater than the 30 cm recommended by the Detection of Environmental *Burkholderia pseudomallei* Working Party (DEBWorP)^21^ and that ranges of spatial auto-correlation between *B. pseudomallei* counts could be higher than previously reported^6^. These outcomes have important implications for the detection of environmental *B. pseudomallei*, as they suggest that, in pedological contexts similar to that investigated in this study, a square sampling grid of 16 × 5 m quadrats (i.e. 20 × 20 m) to 25 × 5 m squares (i.e. 25 × 25 m) should be amply sufficient to detect *B. pseudomallei* with confidence at one given location, provided that samples are taken at a soil depth of at least 60 cm. Such a recommendation should prove all the more relevant now that more reliable detection methods such as qPCR following culture enrichment are available for routine detection of environmental *B. pseudomallei*. We also believe that future studies of *B. pseudomallei* ecology need to take more account of soil heterogeneity at the micro-scale. Finally, we suggest that more research should focus on the detection of *B. pseudomallei* deep in the soil, down to and in water tables, as, in addition to the findings of this study, previous research in Laos also suggests that particle-bound transport of *B. pseudomallei* from groundwater reservoirs to waterways maybe an important dispersion pathway^18^.

## Methods

### Study site

We selected a flat, rain-fed lowland rice field (GPS coordinates N18°22′51.4″, E102°25′27.8, altitude 180 m above mean sea level) in Nabone village, Phonhong District, Vientiane Province, Laos, that was as close as possible to one of the sites in which *B. pseudomallei* had been detected in a previous study in 1998^23^. Permission to collect samples was obtained from the owner of the field and the village officer. The tropical climate of this region is influenced by the southwest monsoon bringing warm and humid air masses from the Indian Ocean during the wet season (April–September). Rainfall is highly seasonal with more than 80% of annual rainfall occurring during the wet season.

### Soil sampling

Four sampling rounds were conducted: in April 2011 (late dry season); June 2011 (just after the start of the rains but before the field was flooded); November 2011 (at the end of the rainy season); and April 2012 (late dry season). On each occasion the site was divided into 49 squares, subsequently referred to as quadrats, each 5 × 5 metres, marked with stakes and string. A reference marker was left *in situ* to ensure that the squares corresponded exactly on each occasion over the whole duration of the study. In each square, samples of approximately 100 grams were collected at depths of 5, 30 cm, 60 cm and 90 cm using an auger that was washed with distilled water then disinfected with 70% alcohol and dried between the collection of each sample. To assess soil properties in the vicinity of each 100 g sample used for *B. pseudomallei* cultures, an additional soil sample was taken just below, using a standard soil bulk density sampler (100 cm^3^ internal volume steel rings, Eijkelkamp – www.eijkelkamp.com). During the first sampling round, samples were taken from the middle of each quadrat. To reduce the impact of any alteration of the soil structure and ecology caused by the previous sampling, the position of each hole within the square was shifted by 1 metre in subsequent sampling rounds, north, south, east or west according to a random schedule generated with Microsoft Excel. The samples were immediately placed in numbered sterile plastic bags, sealed and placed in a cool box in the shade prior to being transported to the laboratory within 48 hours.

### Soil physicochemical parameters

We systematically measured the water content, bulk density and pH of all the samples collected immediately after each of the four successive sampling rounds. Soil water content and bulk density were measured by collecting an intact soil core using a 100 cm^3^ metal ring; the moist soil core was first weighed, then oven dried at 105 °C for 48 h, then reweighed; soil water content and bulk density are the ratios of water lost after drying/mass of dry soil and mass of dry soil/volume of the core, respectively^53^. For soil pH, 20 g of soil was diluted into 50 ml of water and pH of this solution was subsequently measured with a Hanna (Woonsocket, RI, USA) laboratory pH-meter. In addition, based on the results of cultures, we selected a subset of 58 soil samples from the batch collected in April 2012 to measure a range of soil properties: 22 of these came from a quadrat that had been consistently negative for *B. pseudomallei*, whilst 10, 16, 9 and 1 of these samples came from quadrats that had yielded positive CFU counts on only one (*i.e*. of the four sampling rounds), two (April 2011 and April 2012), three (April, November 2011 and April 2012) and all four sampling rounds respectively (Supplementary Table S2). Soil analyses were carried out at the soil analysis laboratory of the National Institute of Agronomic Research (INRA), Arras, France (http://www6.npc.inra.fr/las). The soil properties measured were:

- Total carbon, nitrogen and organic matter without decarbonation (g/kg)
- C/N ratio
- Cation exchange capacity, Metson method (cmol + /kg)
- Phosphorus (P_2_O_5_), Olsen method (g/kg)

### Microbiological investigations

All soil samples were cultured for *B. pseudomallei* by the same methods used in previous environmental studies undertaken in Laos^23, 47, 50^. Briefly, following receipt in the laboratory, 100 g of each sample was mixed thoroughly by agitation with 100 ml of de-ionised water and then left to sediment overnight at room temperature. The following day, aliquots from the top of the aqueous layer (2 × 10 μl, 2 × 100 μl and 1 × 500 μl) were each spread using a rotary plater onto Ashdown selective agar plates, which were incubated at 40–42 °C in air and inspected daily from day 2 to day 4. A further 1 ml of supernatant was added to 9 ml of a selective enrichment medium consisting of tryptone soya broth containing 4% v/v glycerol, 0.0005% w/v crystal violet, and colistin sulphomethate at a final concentration of 50 mg/l (SBCT-C50 broth). This was incubated at 40–42 °C in air for 48 h, after which 10 μl of surface liquid was plated onto Ashdown agar which was incubated and inspected as above. During the final round of sampling enrichment culture as above was also undertaken using the ‘simplified method’ using threonine-basal salt solution (TBSS) plus colistin 50 mg/l described by Limmathurotsakul *et al*.^46^ Colonies of suspected *B. pseudomallei* were initially identified on the basis of their characteristic colony morphology (purple, flat, dry and wrinkled). Suspect colonies were tested using a highly specific latex agglutination test that employs a monoclonal antibody to the 200 kDa exopolysaccharide of *B. pseudomallei* ^54^. All suspect colonies were also tested for their susceptibility to amoxicillin-clavulanic acid (30 μg) and resistance to colistin (10 μg) by disk diffusion testing on Columbia agar. Isolates that were susceptible to amoxicillin-clavulanic acid (zone of inhibition ≥ 20 mm) and showed no inhibition by colistin were presumptively identified as *B. pseudomallei* if positive by latex agglutination and *B. thailandensis* (a closely related but avirulent soil organism) if negative. 10% of presumptive isolates of each species were confirmed by API 20NE (BioMerieux) and disk diffusion susceptibility testing with amoxicillin-clavulanic acid, ceftazidime, ciprofloxacin, colistin, doxycycline, gentamicin, imipenem, and trimethoprim-sulfamethoxazole by a standard disk diffusion method based on that of the Clinical and Laboratory Standards Institute^55^. *B. thailandensis* gave a profile identical to *B. pseudomallei* but with positive arabinose assimilation. The number of colonies of each species growing on each Ashdown plate was counted and used to estimate the number of colonies per gram (CFUg^−1^) of soil as previously described^23^.

### Data analysis

All analyses were carried out using R version 3.0.2 (R Core Team, 2013). Because most of the data were non-normally distributed, we only used non-parametric tests. We applied the Mann-Whitney-Wilcoxon test (*wilcox_test* function from the *coin* package) to assess whether there were differences between soil properties in positive and negative samples. We tested for differences between soil properties of samples with *B. pseudomallei* present at >100 CFU/g, at 0–100 CFU/g, and negative, using the Kruskal-Wallis test (kruskal.test function from the *stats* package). We also computed Spearman’s rank-order correlation (*rcorr* function from the *Hmisc* package) to measure the strength of the association between the presence of *B. pseudomallei* and some of the measured soil properties.

#### Analysis of *B. pseudomallei* counts spatial distribution

The spatial distributions of *B. pseudomallei* was analysed using empirical semivariograms^56^, which allow assessing the interdependence between *B. pseudomallei* counts and lag distance between sampling points^6^. Comparison between semivariograms of the distributions of *B. pseudomallei* at successive sampling rounds gave some insight into the dynamics of the spatial distribution of *B. pseudomallei* counts. Semivariograms were computed for each sampling round and depth using the *variog* function of the R package *geoR* ^57^. Stationarity was tested by looking for trends in mean and variance of CFU counts along both the North-South and East-West directions. Overall, normality of the data could not be verified because of the prevalence of culture negative sampling points. To reduce the skewness of the distributions of *B. pseudomallei* counts, original CFU numbers to which one was added to avoid having null values, were log transformed prior to computing semivariograms. Variogram models were produced using the *variofit* (Variogram Based Parameter Estimation) and *likfit* (Likelihood Based Parameter Estimation for Gaussian Random Fields) functions from the *geoR* package. We subsequently used these models to estimate the nugget variance, i.e. the variance at distances shorter than the smallest sampling interval, the sill, i.e. the maximum variance value reached by variogram indicative of the absence of auto-correlation and range of spatial auto-correlation which is the lag distance at which the sill is reached. As suggested by Limmathurotsakul *et al*.^6^, we used the nugget/sill ratio to assess the degree of relatedness within spatially separated measurements within the range, with values tending towards 1 and 0, indicating lack of and strong spatial auto-correlation, respectively.

### Data Availability

The datasets generated during and/or analysed during the current study are available from the corresponding author on reasonable request.

## Electronic supplementary material

Burkholderia pseudomallei in a rice paddy supplementary data

## References

[1] Cheng AC, Currie BJ (2005). Melioidosis: epidemiology, pathophysiology, and management. Clin Microbiol Rev.

[2] Inglis TJJ, Mee BJ, Chang BJ (2001). The environmental microbiology of melioidosis. Rev Med Microbiol.

[3] Limmathurotsakul D (2016). Predicted global distribution of *Burkholderia pseudomallei* and burden of melioidosis. Nature Microbiol.

[4] Limmathurotsakul D (2013). Activities of daily living associated with acquisition of melioidosis in northeast Thailand: a matched case-control study. PLoS Negl Trop Dis.

[5] Currie BJ, Jacups SP (2003). Intensity of rainfall and severity of melioidosis, Australia. Emerg Infect Dis.

[6] Limmathurotsakul D (2010). *Burkholderia pseudomallei* is spatially distributed in soil in northeast Thailand. PLoS Negl Trop Dis.

[7] Vuddhakul V (1999). Epidemiology of *Burkholderia pseudomallei* in Thailand. Am J Trop Med Hyg.

[8] Wuthiekanun V (2009). *Burkholderia pseudomallei* is genetically diverse in agricultural land in Northeast Thailand. PLoS Negl Trop Dis.

[9] Draper AD (2010). Association of the melioidosis agent *Burkholderia pseudomallei* with water parameters in rural water supplies in Northern Australia. Appl Environ Microbiol.

[10] Chen YS, Chen SC, Kao CM, Chen YL (2003). Effects of soil pH, temperature and water content on the growth of *Burkholderia pseudomallei*. Folia Microbiol (Praha).

[11] Inglis TJ, Sagripanti JL (2006). Environmental factors that affect the survival and persistence of *Burkholderia pseudomallei*. Appl Environ Microbiol.

[12] Kaestli M (2009). Landscape changes influence the occurrence of the melioidosis bacterium *Burkholderia pseudomallei* in soil in northern Australia. PLoS Negl Trop Dis.

[13] Kaestli M (2012). Out of the ground: aerial and exotic habitats of the melioidosis bacterium *Burkholderia pseudomallei* in grasses in Australia. Environ Microbiol.

[14] Baker AL, Ezzahir J, Gardiner C, Shipton W, Warner JM (2015). Environmental attributes influencing the distribution of *Burkholderia pseudomallei* in Northern Australia. PLoS One.

[15] Hantrakun V (2016). Soil nutrient depletion is associated with the presence of *Burkholderia pseudomallei*. Appl Environ Microbiol.

[16] Musa HI (2016). Physicochemical properties influencing presence of *Burkholderia pseudomallei* in soil from small ruminant farms in peninsular Malaysia. PLoS One.

[17] Ngamsang R (2015). The contribution of soil physicochemical properties to the presence and genetic diversity of *Burkholderia pseudomallei*. Southeast Asian J Trop Med Public Health.

[18] Ribolzi O (2016). Land use and soil type determine the presence of the pathogen *Burkholderia pseudomallei* in tropical rivers. Environ Sci Pollut Res Int.

[19] Sermswan RW, Royros P, Khakhum N, Wongratanacheewin S, Tuanyok A (2015). Direct detection of *Burkholderia pseudomallei* and biological factors in soil. Trans R Soc Trop Med Hyg.

[20] Suebrasri T, Wang-ngarm S, Chareonsudjai P, Sermswan R, Chareonsudjai S (2013). Seasonal variation of soil environmental characteristics affect the presence of *Burkholderia pseudomallei* in Khon Kaen, Thailand. Afr J Microbiol Res.

[21] Limmathurotsakul D (2013). Systematic review and consensus guidelines for environmental sampling of *Burkholderia pseudomallei*. PLoS Negl Trop Dis.

[22] Thomas AD, Forbes-Faulkner J, Parker M (1979). Isolation of *Pseudomonas pseudomallei* from clay layers at defined depths. Am J Epidemiol.

[23] Wuthiekanun V (2005). Detection of *Burkholderia pseudomallei* in soil within the Lao People’s Democratic Republic. J Clin Microbiol.

[24] Phetsouvanh R (2001). Melioidosis and Pandora’s box in the Lao People’s Democratic Republic. Clin Infect Dis.

[25] Vaucel M (1937). Présence probable du bacille de Whitmore dans l’eau de mare au Tonkin. Bull Soc Pathol Exot.

[26] Chambon L (1955). Isolement du bacille de Whitmore à partir du milieu extérieur. Ann Inst Pasteur.

[27] Chantratita N (2008). Genetic diversity and microevolution of *Burkholderia pseudomallei* in the environment. PLoS Negl Trop Dis.

[28] Wuthiekanun V, Smith MD, Dance DA, White NJ (1995). Isolation of *Pseudomonas pseudomallei* from soil in north-eastern Thailand. Trans R Soc Trop Med Hyg.

[29] Kaestli M (2007). Sensitive and specific molecular detection of *Burkholderia pseudomallei*, the causative agent of melioidosis, in the soil of tropical northern Australia. Appl Environ Microbiol.

[30] Kao CM, Chen SC, Chen YS, Lin HM, Chen YL (2003). Detection of *Burkholderia pseudomallei* in rice fields with PCR-based technique. Folia Microbiol (Praha).

[31] Palasatien, S., Lertsirivorakul, R., Royros, P., Wongratanacheewin, S. & Sermswan, R. W. Soil physicochemical properties related to the presence of *Burkholderia pseudomallei*. *Trans R Soc Trop Med Hyg* **102** Suppl 1, S5–9, 10.1016/S0035-9203(08)70003-8 (2008). 10.1016/S0035-9203(08)70003-8 19121688

[32] Modis K, Papaodysseus K (2006). Theoretical estimation of the critical sampling size for homogeneous ore bodies with small nugget effect. Math Geol.

[33] Chandler DP, Brockman FJ, Bailey TJ, Fredrickson JK (1998). Phylogenetic diversity of Archaea and bacteria in a deep subsurface paleosol. Microb Ecol.

[34] Mayo M (2011). *Burkholderia pseudomallei* in unchlorinated domestic bore water, Tropical Northern Australia. Emerg Infect Dis.

[35] Wang-Ngarm S, Chareonsudjai S, Chareonsudjai P (2014). Physicochemical factors affecting the growth of *Burkholderia pseudomallei* in soil microcosm. Am J Trop Med Hyg.

[36] Stopnisek N (2014). Genus-wide acid tolerance accounts for the biogeographical distribution of soil *Burkholderia* populations. Environ Microbiol.

[37] Hill AA (2013). Melioidosis as a consequence of sporting activity. Am J Trop Med Hyg.

[38] Baker A (2011). Groundwater seeps facilitate exposure to *Burkholderia pseudomallei*. Appl Environ Microbiol.

[39] Tong S, Yang S, Lu Z, He W (1996). Laboratory investigation of ecological factors influencing the environmental presence of *Burkholderia pseudomallei*. Microbiol Immunol.

[40] Kanai K, Kondo E (1994). Recent advances in biomedical sciences of *Burkholderia pseudomallei* (basonym: *Pseudomonas pseudomallei*). Jap J Med Sci Biol.

[41] Kamjumphol W, Chareonsudjai P, Taweechaisupapong S, Chareonsudjai S (2015). Morphological alteration and survival of *Burkholderia pseudomallei* in soil microcosms. Am J Trop Med Hyg.

[42] Yang H, Kooi CD, Sokol PA (1993). Ability of *Pseudomonas pseudomallei* malleobactin to acquire transferrin-bound, lactoferrin-bound, and cell-derived iron. Infect Immun.

[43] Kvitko BH, Goodyear A, Propst KL, Dow SW, Schweizer HP (2012). *Burkholderia pseudomallei* known siderophores and hemin uptake are dispensable for lethal murine melioidosis. PLoS Negl Trop Dis.

[44] Brook MD, Currie B, Desmarchelier PM (1997). Isolation and identification of *Burkholderia pseudomallei* from soil using selective culture techniques and the polymerase chain reaction. J Appl Microbiol.

[45] Chen YS (2010). Distribution of melioidosis cases and viable *Burkholderia pseudomallei* in soil: evidence for emerging melioidosis in Taiwan. J Clin Microbiol.

[46] Limmathurotsakul D (2012). Effectiveness of a simplified method for isolation of *Burkholderia pseudomallei* from soil. Appl Environ Microbiol.

[47] Rattanavong S (2011). Randomized soil survey of the distribution of *Burkholderia pseudomallei* in rice fields in Laos. Appl Environ Microbiol.

[48] Thomas AD, Forbes-Faulkner JC (1981). Persistence of *Pseudomonas pseudomallei* in soil. Aust Vet J.

[49] Trung TT (2011). Improved culture-based detection and quantification of *Burkholderia pseudomallei* from soil. Trans R Soc Trop Med Hyg.

[50] Vongphayloth K (2012). *Burkholderia pseudomallei* detection in surface water in southern Laos using Moore’s swabs. Am J Trop Med Hyg.

[51] Trung TT (2011). Highly sensitive direct detection and quantification of *Burkholderia pseudomallei* bacteria in environmental soil samples by using real-time PCR. Appl Environ Microbiol.

[52] Knappik M (2015). Evaluation of molecular methods to improve the detection of *Burkholderia pseudomallei* in soil and water samples from Laos. Appl Environ Microbiol.

[53] McKenzie, N. J., Jacquier, D. J., Isbell, R. F. & Brown, K. L. *Australian Soils and Landscapes. An Illustrated Compendium*. (CSIRO Publishing, 2004).

[54] Wuthiekanun V, Anuntagool N, White NJ, Sirisinha S (2002). A rapid method for the differentiation of *Burkholderia pseudomallei* and *Burkholderia thailandensis*. Am J Trop Med Hyg.

[55] CLSI. Performance Standards for Antimicrobial Susceptibility Testing; Twenty-First Informational Supplement. CLSI document M100-S21. (Clinical and Laboratory Standards Institute, 2011).

[56] Diggle, P. J. & Ribeiro, P. J. *Model-based Geostatistics*. (Springer, 2007).

[57] R: A Language and Environment for Statistical Computing (R Foundation for Statistical Computing, Vienna, Austria, 2014).

