# Supplementary material for: Burkholderia pseudomallei in a lowland rice paddy: seasonal changes and influence of soil depth and physico-chemical properties
Source: Sci Rep. 2017 Jun 8;7:3031. doi: 10.1038/s41598-017-02946-z (PMC5465195; doi:10.1038/s41598-017-02946-z)
Supplement: Supplementary file 1 — Burkholderia pseudomallei in a rice paddy supplementary data [file 41598_2017_2946_MOESM1_ESM.pdf]

Supplementary Data

Burkholderia pseudomallei in a lowland rice paddy: seasonal changes and influence of soil depth and physico-chemical properties

Manivanh L[1,2], Pierret A[3], Rattanavong S[2], Kounnavongsa O[1], Buisson Y[1], Elliott I[2,4], Maeght J-L[5], Xayyathip K[3], Silisouk J[2], Vongsouvath M[2], Phetsouvanh R[2,4+], Newton PN[2,4], Lacombe G[6], Ribolzi O[7], Rochelle-Newall E[8], Dance DAB*[2,4,9]

[1] Institut de la Francophonie pour la Médecine Tropicale, BP 9519, Vientiane, Lao People's Democratic Republic

[2] Lao-Oxford-Mahosot Hospital-Wellcome Trust Research Unit, Microbiology Laboratory, Mahosot Hospital, Vientiane, Lao People's Democratic Republic

[3] iEES-Paris (IRD-Sorbonne Universités -UPMC-CNRS-INRA-UDD-UPEC), c/o Department of Agricultural Land Management (DALaM), Vientiane, Laos

[4] Centre for Tropical Medicine & Global Health, University of Oxford, UK

[5] iEES-Paris (IRD-Sorbonne Universités -UPMC-CNRS-INRA-UDD-UPEC), c/o Soils and Fertilizers Research Institute (SFRI), Hanoi, Vietnam

[6] International Water Management Institute (IWMI), Southeast Asia Regional Office, PO BOX 4199, Vientiane, Lao PDR

[7] Géosciences Environnement Toulouse – GET (IRD, Université de Toulouse, CNES, CNRS, UPS) 31400 Toulouse, France

[8] iEES-Paris (IRD-Sorbonne Universités -UPMC-CNRS-INRA-UDD-UPEC), Université Pierre et Marie-Curie, Paris, France

[9] Faculty of Infectious and Tropical Diseases, London School of Hygiene and Tropical Medicine, London, UK

+ Deceased

*Corresponding author: David Dance

Running title: *Burkholderia pseudomallei* in a Lao rice paddy

Table s.1

B. pseudomallei count (CFU g^{-1}), Soil Bulk Density (Mg m^{-3}), Soil Water Content (g g^{-1}), for each sampling quadrat at each of the four sampling depths and for each sampling round. Missing values are indicated by “NA”.

Table s.2

Soil analyses for a selection of 58 samples. Organic Matter (g kg^{-1}); Total organic C (g kg^{-1} , NF ISO 10694) and Total organic N (g kg^{-1}, NF ISO 13878); C/N; P2O5 (g kg^{-1}, Olsen method; NF ISO 11263); Cation Exchange Capacity – CEC (cmol+ kg^{-1}, Metson method, NF X 31-130).

Table s.3

Soil pH (H_20) for a selection of 98 samples.

Table s.1

				B. pseudomallei count (CFU g^{-1})					Soil Bulk Density (Mg m^{-3})				Soil Water Content (g g^{-1})			
**Sample**	**X (m)**	**Y (m)**	**Depth (cm)**	**Apr-11**	**Jun-11**	**Nov-11**	**Apr-12**	**Positivity**	**Apr-11**	**Jun-11**	**Nov-11**	**Apr-12**	**Apr-11**	**Jun-11**	**Nov-11**	**Apr-12**
1.1	2.5	2.5	5	0	0	0	0	0	1.38	1.64	1.5	1.23	17.22	20.46	24.51	21.15
1.2	2.5	2.5	30	0	6	25	950	3	1.34	1.54	1.4	1.33	23.33	23.96	26.38	27.45
1.3	2.5	2.5	60	20	NA	74	1800	3	1.44	NA	1.42	1.66	24.48	NA	30.28	19.24
1.4	2.5	2.5	90	142	NA	NA	NA	1	1.53	NA	NA	NA	23.31	NA	NA	NA
2.1	2.5	7.5	5	0	0	0	0	0	1.43	1.61	1.31	1.42	15.38	19.88	26.64	20.12
2.2	2.5	7.5	30	10	0	0	0	1	1.52	1.43	1.3	1.46	21.39	26.82	31.75	22.21
2.3	2.5	7.5	60	25	NA	0	230	2	1.55	NA	1.5	1.47	25.31	NA	27.06	26.45
2.4	2.5	7.5	90	25	NA	NA	180	2	1.28	NA	NA	1.4	29.11	NA	NA	27.97
3.1	2.5	12.5	5	0	0	20	0	0	1.55	1.56	1.47	1.45	17.32	19.94	24.31	19.31
3.2	2.5	12.5	30	0	1358	65	0	2	1.38	1.51	1.44	1.48	18.71	24.26	26.92	23.76
3.3	2.5	12.5	60	400	NA	420	10	2	1.59	NA	1.5	1.45	25.2	NA	24.35	25.07
3.4	2.5	12.5	90	3980	NA	NA	70	2	1.58	NA	NA	1.15	24.02	NA	NA	24.45
4.1	2.5	17.5	5	0	0	0	1	1	1.75	1.63	1.38	1.48	12.17	18.83	26.36	20.89
4.2	2.5	17.5	30	0	0	83	0	1	1.57	1.71	1.53	1.51	20.42	24.39	25.78	16.47
4.3	2.5	17.5	60	75	NA	295	0	2	1.69	NA	1.59	1.56	22.19	NA	24.05	22.12
4.4	2.5	17.5	90	140	NA	NA	650	2	1.56	NA	NA	1.58	24.67	NA	NA	24.46
5.1	2.5	22.5	5	0	0	0	0	0	1.5	1.42	1.26	1.45	17.74	25.86	29.35	23.55
5.2	2.5	22.5	30	0	0	0	0	0	1.53	1.63	1.53	1.5	16.82	20.01	24.59	24.12
5.3	2.5	22.5	60	2	NA	100	100	3	1.51	NA	1.5	1.54	24.35	NA	25.14	21.76
5.4	2.5	22.5	90	0	NA	NA	0	0	1.59	NA	NA	1.54	23.27	NA	NA	20.1
6.1	2.5	27.5	5	0	0	0	0	0	1.7	1.23	1.36	1.24	17.09	30	31.23	25.78
6.2	2.5	27.5	30	0	35	7	50	3	1.64	1.46	1.49	1.56	21.5	31.81	22.39	21.25
6.3	2.5	27.5	60	10	NA	265	600	3	1.71	NA	1.62	1.55	17.83	NA	21.94	20.25
6.4	2.5	27.5	90	50	NA	NA	70	2	2.17	NA	NA	1.64	5.02	NA	NA	18.09
7.1	2.5	32.5	5	0	0	0	0	0	1.6	1.41	1.19	1.43	16.02	27.8	30.74	21.15
7.2	2.5	32.5	30	1	20	0	0	2	1.47	1.66	1.48	1.46	20.21	17.95	19.98	22.49
7.3	2.5	32.5	60	0	NA	5	0	1	1.69	NA	1.7	1.68	18.48	NA	22.39	20.62
7.4	2.5	32.5	90	1	NA	NA	0	1	1.65	NA	NA	1.55	18.53	NA	NA	21.24
8.1	7.5	2.5	5	0	0	0	0	0	1.46	1.46	1.45	1.67	17.9	24.86	28.08	15.15
8.2	7.5	2.5	30	275	3100	12	150	4	1.51	1.45	1.53	1.45	17.53	26.77	23.82	19.86
8.3	7.5	2.5	60	325	NA	218	410	3	1.57	NA	1.54	1.46	20.52	NA	24.63	25.39
8.4	7.5	2.5	90	0	NA	NA	850	1	1.56	NA	NA	1.68	24.26	NA	NA	22.9
9.1	7.5	7.5	5	1	0	0	1	2	1.63	1.51	1.47	1.53	15.77	26.78	24.7	18.75
9.2	7.5	7.5	30	0	0	90	0	1	1.52	1.59	1.36	1.55	20.47	22.25	27.55	19.47
9.3	7.5	7.5	60	15	NA	5060	0	2	1.53	NA	1.53	1.38	22.87	NA	23.43	29.43
9.4	7.5	7.5	90	362	NA	NA	500	2	1.28	NA	NA	1.43	30.42	NA	NA	22.7
10.1	7.5	12.5	5	0	0	0	0	0	1.52	1.35	1.41	1.3	17.87	27.15	26.12	22.25
10.2	7.5	12.5	30	0	0	0	0	0	1.57	1.47	1.56	1.53	21.7	23.88	24.66	21.25

10.3	7.5	12.5	60	0	0	0	600	1	1.46	1.61	1.57	1.47	26.18	21.74	23.07	27.87
10.4	7.5	12.5	90	690	NA	NA	0	1	1.36	NA	NA	1.21	32.39	NA	NA	29.77
11.1	7.5	17.5	5	0	0	0	0	0	1.39	1.68	1.41	1.33	13.3	19.34	28.61	19.02
11.2	7.5	17.5	30	5	0	0	10	2	1.51	1.61	1.58	1.43	18.06	21.41	21.65	22.25
11.3	7.5	17.5	60	0	NA	73	65	2	1.47	NA	1.34	1.5	21.63	NA	30.63	24.31
11.4	7.5	17.5	90	0	NA	NA	10	1	1.24	NA	NA	NA	36.34	NA	NA	NA
12.1	7.5	22.5	5	0	0	0	0	0	1.72	1.47	1.4	1.24	16.38	23.19	27.16	22.48
12.2	7.5	22.5	30	0	0	0	50	1	1.55	1.46	1.55	1.59	19.42	24.56	23.87	20.51
12.3	7.5	22.5	60	127	NA	6778	300	3	1.52	NA	1.32	1.25	24.12	NA	30.35	28.69
12.4	7.5	22.5	90	5	NA	NA	1400	2	1.3	NA	NA	NA	27.56	NA	NA	NA
13.1	7.5	27.5	5	0	0	0	0	0	1.42	1.42	1.31	1.47	20.86	26.07	31.44	20.86
13.2	7.5	27.5	30	0	0	0	0	0	1.6	1.57	1.48	1.48	20.59	22.26	22.81	22.71
13.3	7.5	27.5	60	0	NA	0	125	1	1.46	NA	1.44	1.51	27.56	NA	27.55	23.41
13.4	7.5	27.5	90	50	NA	0	1	2	1.46	NA	1.49	1.41	24.01	NA	27.29	25.74
14.1	7.5	32.5	5	0	0	0	0	0	1.66	1.4	1.28	1.37	18.29	27.57	31.44	26.15
14.2	7.5	32.5	30	0	0	0	0	0	1.52	1.64	1.45	1.57	21.37	21.45	21.78	19.3
14.3	7.5	32.5	60	375	NA	0	0	1	1.63	NA	1.53	1.62	20.38	NA	26.82	21.08
14.4	7.5	32.5	90	0	NA	NA	0	0	1.7	NA	NA	1.55	19.28	NA	NA	21.07
15.1	12.5	2.5	5	0	5	0	0	1	1.62	1.45	1.51	1.49	15.68	26.9	25.38	19.52
15.2	12.5	2.5	30	0	0	0	30	1	1.47	1.5	1.47	1.49	23.34	23.78	22.63	22.28
15.3	12.5	2.5	60	0	NA	0	0	0	1.58	NA	1.51	1.29	22.85	NA	24.95	28.63
15.4	12.5	2.5	90	1	NA	NA	NA	1	1.31	NA	NA	NA	34.77	NA	NA	NA
16.1	12.5	7.5	5	0	0	0	0	0	1.71	1.34	1.41	1.39	17.02	25.95	28.83	20.39
16.2	12.5	7.5	30	0	190	0	0	1	1.62	1.59	1.54	1.51	18.22	22.95	24.74	21.99
16.3	12.5	7.5	60	0	NA	39	0	1	1.54	NA	1.58	1.49	22.56	NA	24.62	23.68
16.4	12.5	7.5	90	1	NA	NA	NA	1	1.32	NA	NA	NA	34.25	NA	NA	NA
17.1	12.5	12.5	5	0	2500	0	0	1	1.57	1.47	1.21	1.33	17.42	24.44	26.22	18.31
17.2	12.5	12.5	30	0	0	0	0	0	1.62	1.55	1.51	1.04	19.42	22.78	23.86	15.59
17.3	12.5	12.5	60	75	NA	0	0	1	1.63	NA	1.3	1.32	21.47	NA	36.32	24.02
17.4	12.5	12.5	90	100	NA	NA	0	1	1.55	NA	NA	1.47	24.32	NA	NA	23.34
18.1	12.5	17.5	5	20	0	0	0	1	1.69	1.65	1.42	1.33	15.16	17.88	27.53	21.77
18.2	12.5	17.5	30	0	0	0	0	0	1.46	1.55	1.5	1.61	21.67	21.3	23.38	20.48
18.3	12.5	17.5	60	40	0	0	0	1	1.4	NA	1.39	1.45	28.73	NA	32.42	24.13
18.4	12.5	17.5	90	400	NA	NA	1	2	1.56	NA	NA	NA	21.18	NA	NA	NA
19.1	12.5	22.5	5	0	0	0	0	0	1.53	1.33	1.18	1.4	21.06	28.5	29.6	18.81
19.2	12.5	22.5	30	0	0	0	450	1	1.52	1.54	1.49	1.48	21.1	21.41	24.88	21.63
19.3	12.5	22.5	60	0	4	52	450	3	1.32	1.56	1.49	1.49	24.83	23.84	25.46	26.12
19.4	12.5	22.5	90	145	NA	NA	100	2	1.28	NA	NA	1.46	32.79	NA	NA	24.29
20.1	12.5	27.5	5	0	0	0	0	0	1.61	1.45	1.42	1.39	17.04	23.96	30.59	19.71
20.2	12.5	27.5	30	34	0	0	0	1	1.6	1.47	1.45	1.54	19.99	25.66	26.44	19.47
20.3	12.5	27.5	60	393	NA	106	0	2	1.49	NA	1.33	1.26	24.92	NA	29.82	26.47
20.4	12.5	27.5	90	20	NA	NA	0	1	1.52	NA	NA	1.28	24.33	NA	NA	27.81
21.1	12.5	32.5	5	0	0	128	0	1	1.65	1.5	1.3	1.43	17.17	22.84	29.11	21.34
21.2	12.5	32.5	30	1	1	0	0	2	1.6	1.71	1.61	1.49	17.96	17.65	21.05	19

21.3	12.5	32.5	60	110	405	83	0	3	1.44	1.44	1.3	1.38	27.17	29.57	39.91	31
21.4	12.5	32.5	90	55	NA	NA	0	1	1.47	NA	NA	1.42	27.77	NA	NA	24.45
22.1	17.5	2.5	5	0	0	0	1350	1	1.56	1.54	1.44	1.36	17.61	22.73	26.2	17.19
22.2	17.5	2.5	30	0	0	0	200	1	1.58	1.5	1.52	1.48	21.37	24.53	23.36	23.28
22.3	17.5	2.5	60	0	0	0	300	1	1.58	NA	1.6	1.16	22.02	NA	22.92	23.88
22.4	17.5	2.5	90	60	NA	NA	2750	2	1.58	NA	NA	1.44	24.37	NA	NA	27.3
23.1	17.5	7.5	5	0	0	0	0	0	1.64	1.42	1.38	1.51	17.49	24.53	27.74	19.33
23.2	17.5	7.5	30	0	490	2	0	2	1.46	1.4	1.43	1.35	26.01	29.09	28.5	21.01
23.3	17.5	7.5	60	0	NA	0	680	1	1.47	NA	1.69	1.22	24.75	NA	21.59	22.04
23.4	17.5	7.5	90	0	NA	NA	0	0	1.63	NA	NA	1.36	20.78	NA	NA	21.85
24.1	17.5	12.5	5	0	0	23	0	1	1.49	1.58	1.34	1.38	16.52	24.68	30.56	18.6
24.2	17.5	12.5	30	0	0	0	0	0	1.56	1.5	1.53	1.19	18.3	23.4	20.29	16.48
24.3	17.5	12.5	60	4	NA	13	0	2	1.58	NA	1.58	1.57	22.82	NA	24.92	21.44
24.4	17.5	12.5	90	35	NA	NA	0	1	1.56	NA	NA	1.36	21.2	NA	NA	30.12
25.1	17.5	17.5	5	0	0	0	0	0	1.62	1.4	1.26	1.4	15.15	23.6	28.84	19.94
25.2	17.5	17.5	30	1	0	0	0	1	1.53	1.42	1.26	1.53	20.48	26.84	24.03	20.12
25.3	17.5	17.5	60	550	NA	0	50	2	1.39	NA	1.41	1.39	30.76	NA	27.5	28.07
25.4	17.5	17.5	90	850	NA	NA	4050	2	1.42	NA	NA	NA	30.02	NA	NA	NA
26.1	17.5	22.5	5	0	0	36	0	1	1.59	1.35	1.35	1.38	16.72	27.45	33.25	22.88
26.2	17.5	22.5	30	250	0	5	0	2	1.56	1.53	1.49	1.55	22.82	23.14	25.7	19.99
26.3	17.5	22.5	60	0	NA	22	0	1	1.24	NA	1.49	1.51	30.6	NA	26.24	23.36
26.4	17.5	22.5	90	1	NA	NA	0	1	1.3	NA	NA	1.5	28.98	NA	NA	22.32
27.1	17.5	27.5	5	0	0	0	0	0	1.56	1.36	1.3	1.46	20.46	27.63	31.41	21.67
27.2	17.5	27.5	30	0	0	0	100	1	1.63	1.32	1.36	1.3	19.81	28.72	26.24	19.71
27.3	17.5	27.5	60	0	NA	0	15	1	1.54	NA	1.37	1.47	24.17	NA	28.39	24.27
27.4	17.5	27.5	90	45	NA	NA	250	2	1.32	NA	NA	1.04	27.54	NA	NA	27.09
28.1	17.5	32.5	5	0	0	0	0	0	1.58	1.54	1.27	1.51	16.23	26.25	31.57	20.43
28.2	17.5	32.5	30	20	0	0	0	1	1.41	1.51	1.4	1.38	24.37	23.92	25.29	26.67
28.3	17.5	32.5	60	14	0	0	0	1	1.62	1.46	1.35	1.45	20.77	27.44	30.88	24.03
28.4	17.5	32.5	90	20	NA	NA	100	2	1.56	NA	NA	1.56	23.85	NA	NA	22.15
29.1	22.5	2.5	5	0	0	0	0	0	1.64	1.58	1.36	1.55	16.64	22.22	26.5	18.7
29.2	22.5	2.5	30	0	0	0	0	0	1.67	1.62	1.56	1.46	20.14	21.57	24.45	21.37
29.3	22.5	2.5	60	0	0	0	0	0	1.59	1.63	1.59	1.62	22.17	23.5	24.24	21.85
29.4	22.5	2.5	90	0	NA	NA	0	0	1.58	NA	NA	1.51	23.15	NA	NA	23.51
30.1	22.5	7.5	5	0	0	0	0	0	1.5	1.43	1.44	1.33	21.12	25.44	23.58	20.38
30.2	22.5	7.5	30	0	0	0	0	0	1.57	1.52	1.56	1.52	21	24.37	22.93	21.77
30.3	22.5	7.5	60	0	0	0	0	0	1.59	1.54	1.63	1.49	22.64	23.78	23.53	22.71
30.4	22.5	7.5	90	15	NA	NA	250	2	1.64	NA	NA	1.38	22.52	NA	NA	23.44
31.1	22.5	12.5	5	0	145	0	0	1	1.61	1.53	1.33	1.34	17.67	22.22	29.71	17.97
31.2	22.5	12.5	30	27	1	10	200	4	1.55	1.61	1.55	1.45	21.13	23.79	22.69	17.8
31.3	22.5	12.5	60	15	240	0	1	3	1.61	1.66	1.6	1.53	21.96	21.57	24.31	22.72
31.4	22.5	12.5	90	0	NA	NA	0	0	1.68	NA	NA	1.55	22.43	NA	NA	22.81
32.1	22.5	17.5	5	0	0	0	0	0	1.26	1.49	1.36	1.18	23.52	24.38	26.85	20.19
32.2	22.5	17.5	30	0	0	5	1	2	1.63	1.47	1.62	1.51	18.01	28.39	21.52	17.63

32.3	22.5	17.5	60	0	NA	0	0	0	1.43	NA	1.29	1.48	26.87	NA	34.86	30.35
32.4	22.5	17.5	90	0	NA	NA	0	0	1.53	NA	NA	1.41	25.99	NA	NA	28.29
33.1	22.5	22.5	5	0	0	0	0	0	1.6	1.4	1.31	1.48	18.78	21.8	29.48	20.39
33.2	22.5	22.5	30	4	6	0	0	2	1.61	1.47	1.19	1.51	20.66	21.66	22.71	20.07
33.3	22.5	22.5	60	0	NA	35	0	0	1.56	NA	1.35	1.04	22.93	NA	35.44	28.09
33.4	22.5	22.5	90	0	NA	NA	0	0	1.48	NA	NA	NA	25.67	NA	NA	NA
34.1	22.5	27.5	5	0	0	5	0	1	1.65	1.37	1.2	1.29	17.14	24.81	28.61	21.43
34.2	22.5	27.5	30	0	0	0	0	0	1.51	1.47	1.38	1.45	23.62	23.8	21.16	27.19
34.3	22.5	27.5	60	0	NA	0	8150	1	1.53	NA	1.52	1.58	23.81	NA	26.71	22.32
34.4	22.5	27.5	90	0	NA	NA	1200	1	1.59	NA	NA	1.29	23.01	NA	NA	23.69
35.1	22.5	32.5	5	0	1130	0	0	1	1.53	1.32	1.39	1.69	20.7	26.7	27.06	20.97
35.2	22.5	32.5	30	0	955	1	0	1	1.41	1.33	1.39	1.5	25.84	24.61	25.37	23.49
35.3	22.5	32.5	60	0	2175	2	0	2	1.55	1.61	1.55	1.52	24.67	20.8	25.2	22.8
35.4	22.5	32.5	90	0	NA	0	0	0	1.49	NA	1.6	1.32	23.35	NA	22.84	22.14
36.1	27.5	2.5	5	0	0	0	0	0	1.67	1.61	1.5	1.65	16.61	19.77	24.93	16.07
36.2	27.5	2.5	30	0	0	0	0	0	1.58	1.51	1.45	1.42	20.36	23.09	23.8	20.1
36.3	27.5	2.5	60	0	0	0	0	0	1.61	1.53	1.59	1.46	21.83	24.23	25.21	22.75
36.4	27.5	2.5	90	0	NA	NA	0	0	1.6	NA	NA	1.48	23.97	NA	NA	21.06
37.1	27.5	7.5	5	0	0	30	0	1	1.68	1.52	1.35	1.29	17.33	23.75	31.28	14.32
37.2	27.5	7.5	30	0	0	5	0	1	1.57	1.47	1.48	1.49	20.15	24.93	27.86	20.3
37.3	27.5	7.5	60	0	0	0	50	1	1.52	1.56	1.7	1.33	21.41	23.3	23.51	23.57
37.4	27.5	7.5	90	0	NA	NA	0	0	1.62	NA	NA	1.58	22.82	NA	NA	22.35
38.1	27.5	12.5	5	25	0	0	0	1	1.41	1.43	1.35	1.45	25.44	27.39	29.94	18.18
38.2	27.5	12.5	30	0	0	0	0	0	1.52	1.56	1.57	1.55	22.21	21.02	25.98	20.99
38.3	27.5	12.5	60	0	0	0	0	0	1.61	1.61	1.66	1.49	22.11	21.82	26.88	22.59
38.4	27.5	12.5	90	5	NA	NA	0	1	1.62	NA	NA	1.65	21.67	NA	NA	23.88
39.1	27.5	17.5	5	0	0	0	0	0	1.49	1.43	1.3	1.46	23.15	24.07	30.21	18.11
39.2	27.5	17.5	30	0	0	38	0	1	1.62	1.47	1.48	1.47	19.23	22.54	25.17	20.04
39.3	27.5	17.5	60	0	0	0	0	0	1.55	NA	1.5	1.63	22.97	NA	30.64	21.4
39.4	27.5	17.5	90	0	NA	NA	NA	0	1.61	NA	NA	NA	23.43	NA	NA	NA
40.1	27.5	22.5	5	0	1	0	0	1	1.4	1.36	1.34	1.38	25.48	26.76	27.93	20.96
40.2	27.5	22.5	30	0	0	0	0	0	1.64	1.54	1.55	1.55	19.28	19.29	24.81	19.17
40.3	27.5	22.5	60	0	0	0	10	1	1.46	1.49	1.51	1.35	26.72	23.11	26.7	26.54
40.4	27.5	22.5	90	0	NA	NA	0	0	1.39	NA	NA	1.54	31.93	NA	NA	22.42
41.1	27.5	27.5	5	0	0	0	0	0	1.58	1.36	1.17	1.17	18.48	27.87	32.73	20.54
41.2	27.5	27.5	30	0	0	0	450	1	1.5	1.55	1.41	1.46	24.87	21.43	28.01	23.81
41.3	27.5	27.5	60	14	NA	0	0	1	1.5	NA	1.42	1.42	22.96	NA	28.71	26.75
41.4	27.5	27.5	90	0	NA	NA	0	0	1.53	NA	NA	1.39	24.65	NA	NA	23.14
42.1	27.5	32.5	5	0	0	0	0	0	1.36	1.37	1.44	1.4	30.8	25.91	26.14	24.61
42.2	27.5	32.5	30	0	0	0	0	0	1.38	1.55	1.43	1.21	26.7	24.51	25.3	25.95
42.3	27.5	32.5	60	0	0	0	25	1	1.58	1.54	1.64	1.41	25.11	19.3	22.92	23.51
42.4	27.5	32.5	90	0	NA	NA	100	1	1.61	NA	NA	1.59	21.48	NA	NA	19.6
43.1	32.5	2.5	5	0	0	0	0	0	1.36	1.68	1.44	1.27	22.04	20.57	25.64	19.09
43.2	32.5	2.5	30	45	0	26	0	2	1.62	1.61	1.5	1.79	20.84	22.54	25.09	18.99

43.3	32.5	2.5	60	0	0	182	0	1	1.64	1.49	1.65	1.45	20.92	24.93	24.25	21.69
43.4	32.5	2.5	90	0	NA	NA	400	1	1.59	NA	NA	1.36	22.19	NA	NA	22.57
44.1	32.5	7.5	5	0	0	0	0	0	1.63	1.37	1.45	1.36	17.22	23.38	27.19	19.29
44.2	32.5	7.5	30	0	0	4	0	1	1.64	1.5	1.52	1.4	17.93	22.48	25.59	21.26
44.3	32.5	7.5	60	0	NA	250	50	2	1.68	NA	1.55	1.56	17.52	NA	26.6	22.57
44.4	32.5	7.5	90	0	NA	NA	0	0	1.71	NA	NA	1.51	15.79	NA	NA	24.89
45.1	32.5	12.5	5	0	0	8	1	1	1.66	1.45	1.44	1.23	17.49	24.52	28.59	18.62
45.2	32.5	12.5	30	0	0	0	1	1	1.53	1.56	1.63	1.59	21.13	25.3	22.34	19.95
45.3	32.5	12.5	60	0	NA	0	100	1	1.65	NA	1.5	1.49	16.94	NA	28.11	21.69
45.4	32.5	12.5	90	0	NA	NA	10	1	1.62	NA	NA	1.45	19.76	NA	NA	22.83
46.1	32.5	17.5	5	0	50	0	1	2	1.51	1.48	1.32	1.27	18.99	24.17	30.32	18.3
46.2	32.5	17.5	30	200	50	0	0	2	1.53	1.54	1.48	1.26	18.63	22.17	23.43	19.38
46.3	32.5	17.5	60	10	0	1	0	1	1.65	1.53	1.54	1.36	18.29	21.63	22.11	28.94
46.4	32.5	17.5	90	70	NA	NA	0	1	1.64	NA	NA	1.35	21.2	NA	NA	25.85
47.1	32.5	22.5	5	0	0	0	0	0	1.63	1.39	1.52	1.41	19.05	27.14	31.27	18.46
47.2	32.5	22.5	30	1	0	15	0	2	1.63	1.34	1.44	1.75	18.89	28.05	29.85	18.74
47.3	32.5	22.5	60	0	NA	50	0	1	1.65	NA	1.49	1.14	18.66	NA	24.87	23.12
47.4	32.5	22.5	90	31	NA	NA	400	2	1.69	NA	NA	1.23	20.2	NA	NA	20.51
48.1	32.5	27.5	5	0	0	5	1	1	1.38	1.35	1.34	1.42	27.3	24.82	30.85	22.18
48.2	32.5	27.5	30	0	0	0	0	0	1.63	1.46	1.46	1.53	17.16	22.71	26.2	19.69
48.3	32.5	27.5	60	0	NA	0	0	0	1.54	NA	1.21	1.5	22.83	NA	32.66	23.82
48.4	32.5	27.5	90	0	NA	NA	0	0	1.42	NA	NA	1.44	30.1	NA	NA	23.04
49.1	32.5	32.5	5	0	0	0	0	0	1.43	1.38	1.32	1.55	26.19	24.04	30.41	23.84
49.2	32.5	32.5	30	0	0	0	0	0	1.44	1.46	1.34	1.79	24.82	28.62	27.23	23.99
49.3	32.5	32.5	60	0	NA	0	0	0	1.59	NA	1.66	1.44	24.22	NA	22.71	23.57
49.4	32.5	32.5	90	0	NA	NA	1550	1	1.6	NA	NA	1.52	23.51	NA	NA	21.5

Table s.2

Sample	X (m)	Y (m)	Depth (cm)	Positivity	OM	C Total	N Total	C/N	P_2O_5	CEC
1.1	2.5	2.5	5	0	16.6	9.6	0.855	11.2	0.009	5.23
1.2	2.5	2.5	30	3	4.57	2.64	0.319	8.3	0.0049	5.54
1.3	2.5	2.5	60	3	1.59	0.919	0.107	8.63	0.0049	2.7
1.4	2.5	2.5	90	1	1.81	1.05	0.127	8.24	0.0049	3.95
2.4	2.5	7.5	90	2	3.69	2.13	0.245	8.69	0.0049	5.29
3.4	2.5	12.5	90	2	2.67	1.54	0.183	8.45	0.0049	4.44
4.4	2.5	17.5	90	2	2.57	1.49	0.156	9.51	0.0049	3.41
5.1	2.5	22.5	5	0	15	8.69	0.786	11.1	0.006	4.79
5.2	2.5	22.5	30	0	3.12	1.8	0.216	8.34	0.0049	3.92
5.3	2.5	22.5	60	3	1.82	1.05	0.124	8.46	0.0049	2.65
5.4	2.5	22.5	90	0	1.66	0.957	0.102	9.39	0.0049	2.48
6.1	2.5	27.5	5	0	18.4	10.6	1.08	9.84	0.008	5.8
6.2	2.5	27.5	30	3	2.49	1.44	0.173	8.33	0.0049	3.59
6.3	2.5	27.5	60	3	2.53	1.46	0.145	10.1	0.0049	2.48
6.4	2.5	27.5	90	2	0.831	0.48	0.019	25.3	0.006	1.49
8.1	7.5	2.5	5	0	13.9	8.03	0.728	11	0.0049	5.26
8.2	7.5	2.5	30	4	7.65	4.42	0.512	8.63	0.0049	7.32
8.3	7.5	2.5	60	3	2.89	1.67	0.205	8.14	0.0049	3.69
8.4	7.5	2.5	90	1	1.54	0.888	0.0947	9.38	0.0049	1.79
9.4	7.5	7.5	90	2	1.45	0.836	0.1	8.33	0.0049	2.23
12.1	7.5	22.5	5	0	16.9	9.78	0.996	9.82	0.007	5.69
12.2	7.5	22.5	30	1	6.13	3.54	0.387	9.14	0.0049	6.05
12.3	7.5	22.5	60	3	8.26	4.78	0.572	8.35	0.0049	12.7
12.4	7.5	22.5	90	2	8.51	4.92	0.515	9.56	0.0049	11.8
13.1	7.5	27.5	5	0	16.6	9.59	0.946	10.1	0.009	5.34
13.2	7.5	27.5	30	0	4.92	2.84	0.33	8.6	0.0049	5.08
13.3	7.5	27.5	60	1	1.95	1.13	0.14	8.06	0.0049	3.41
13.4	7.5	27.5	90	2	1.96	1.14	0.134	8.47	0.0049	3.53
18.4	12.5	17.5	90	2	7.97	4.61	0.685	6.73	0.0049	16.7
19.1	12.5	22.5	5	0	16	9.24	0.888	10.4	0.011	5.54
19.2	12.5	22.5	30	1	7.75	4.48	0.49	9.15	0.0049	7.03
19.3	12.5	22.5	60	3	4.26	2.46	0.349	7.04	0.0049	7.92
19.4	12.5	22.5	90	2	3.04	1.76	0.253	6.94	0.0049	5.98
21.3	12.5	32.5	60	3	6.46	3.73	0.477	7.83	0.0049	11.8
22.1	17.5	2.5	5	1	14.9	8.58	0.83	10.3	0.01	4.91
22.2	17.5	2.5	30	1	7.66	4.43	0.427	10.4	0.007	6.36
22.3	17.5	2.5	60	1	5.39	3.11	0.366	8.51	0.0049	8.43
22.4	17.5	2.5	90	2	3.74	2.16	0.271	7.98	0.0049	5.87
25.4	17.5	17.5	90	2	8.64	4.99	0.776	6.44	0.0049	19
27.4	17.5	27.5	90	2	5.19	3	0.383	7.84	0.0049	8.16
28.4	17.5	32.5	90	2	1.72	0.994	0.125	7.96	0.0049	3.04
29.1	22.5	2.5	5	0	12	6.95	0.634	11	0.011	4.77
29.2	22.5	2.5	30	0	2.87	1.66	0.221	7.51	0.0049	4.78
29.3	22.5	2.5	60	0	2.52	1.46	0.204	7.15	0.0049	4.54
29.4	22.5	2.5	90	0	2.46	1.42	0.19	7.45	0.0049	4.25
30.1	22.5	7.5	5	0	13.3	7.68	0.712	10.8	0.011	4.47
30.2	22.5	7.5	30	0	3.52	2.04	0.261	7.81	0.0049	5.22
30.3	22.5	7.5	60	0	2.4	1.39	0.198	7.03	0.0049	4.59
30.4	22.5	7.5	90	2	2.14	1.23	0.169	7.3	0.0049	3.87
31.2	22.5	12.5	30	4	7.24	4.18	0.469	8.92	0.006	6.1
36.1	27.5	2.5	5	0	9.69	5.6	0.504	11.1	0.01	4.33
36.2	27.5	2.5	30	0	3.48	2.01	0.261	7.71	0.006	5.47
36.3	27.5	2.5	60	0	2.63	1.52	0.221	6.89	0.0049	5.06
36.4	27.5	2.5	90	0	2	1.16	0.161	7.19	0.0049	4.22
47.1	32.5	22.5	5	0	17.8	10.3	1	10.3	0.007	6.2
47.2	32.5	22.5	30	2	6.97	4.03	0.409	9.84	0.0049	7.01
47.3	32.5	22.5	60	1	6.31	3.65	0.38	9.59	0.0049	8.3
47.4	32.5	22.5	90	2	1.42	0.822	0.0903	9.1	0.0049	2.42

Table s.3

Sample	X (m)	Y (m)	Depth	Positivity	pH
1.1	2.5	2.5	5	0	5.58
1.3	2.5	2.5	60	3	3.78
1.4	2.5	2.5	90	1	4.87
2.1	2.5	7.5	5	0	4.2
2.2	2.5	7.5	30	1	4.63
3.1	2.5	12.5	5	0	4.06
3.2	2.5	12.5	30	2	4.1
3.3	2.5	12.5	60	2	4.09
3.4	2.5	12.5	90	2	4.55
4.1	2.5	17.5	5	1	5.02
4.2	2.5	17.5	30	1	4.53
4.3	2.5	17.5	60	2	4.81
4.4	2.5	17.5	90	2	5.5
5.2	2.5	22.5	30	0	4.82
5.3	2.5	22.5	60	3	4.29
6.1	2.5	27.5	5	0	4.91
6.3	2.5	27.5	60	3	4.46
6.4	2.5	27.5	90	2	5.08
8.2	7.5	2.5	30	4	4.99
8.3	7.5	2.5	60	3	4.43
9.1	7.5	7.5	5	2	4.65
9.3	7.5	7.5	60	2	3.86
9.4	7.5	7.5	90	2	4.58
11.1	7.5	17.5	5	0	4.61
11.3	7.5	17.5	60	2	3.77
12.1	7.5	22.5	5	0	5.12
12.2	7.5	22.5	30	1	3.91
12.3	7.5	22.5	60	3	4.19
12.4	7.5	22.5	90	2	4.54
13.1	7.5	27.5	5	0	3.68
13.2	7.5	27.5	30	0	4.09
13.3	7.5	27.5	60	1	4.14
14.3	7.5	32.5	60	1	4.74
15.1	12.5	2.5	5	1	4.17
18.1	12.5	17.5	5	1	4.18
19.1	12.5	22.5	5	0	4.65
19.2	12.5	22.5	30	1	3.52
19.3	12.5	22.5	60	3	3.64
19.4	12.5	22.5	90	2	4.73
20.1	12.5	27.5	5	0	3.76
20.2	12.5	27.5	30	1	3.55
20.3	12.5	27.5	60	2	3.38
21.1	12.5	32.5	5	1	3.64
21.2	12.5	32.5	30	2	3.6
21.3	12.5	32.5	60	3	3.54
22.2	17.5	2.5	30	1	4.65
22.3	17.5	2.5	60	1	4.2
23.1	17.5	7.5	5	0	3.74
23.2	17.5	7.5	30	2	3.83
25.1	17.5	17.5	5	0	4.64
25.4	17.5	17.5	90	2	4.68
26.1	17.5	22.5	5	1	4.28
26.2	17.5	22.5	30	2	3.76
28.2	17.5	32.5	30	1	3.87
29.1	22.5	2.5	5	0	4.8
29.3	22.5	2.5	60	0	4.1
29.4	22.5	2.5	90	0	4.68
30.2	22.5	7.5	30	0	4.64
30.3	22.5	7.5	60	0	4.36
31.1	22.5	12.5	5	1	4.64
31.2	22.5	12.5	30	4	4.17
31.3	22.5	12.5	60	3	4.03
33.3	22.5	22.5	60	0	3.85
34.2	22.5	27.5	30	0	4.16

34.3	22.5	27.5	60	1	4.29
35.1	22.5	32.5	5	1	4.17
35.2	22.5	32.5	30	1	3.92
36.1	27.5	2.5	5	0	4.75
36.2	27.5	2.5	30	0	4.38
36.3	27.5	2.5	60	0	4.24
36.4	27.5	2.5	90	0	4.71
37.2	27.5	7.5	30	1	4.23
37.3	27.5	7.5	60	1	4.25
38.1	27.5	12.5	5	1	4.42
38.2	27.5	12.5	30	0	4.04
38.3	27.5	12.5	60	0	4.29
39.2	27.5	17.5	30	1	3.9
39.3	27.5	17.5	60	0	4.37
39.4	27.5	17.5	90	0	4.5
40.1	27.5	22.5	5	1	4.17
40.2	27.5	22.5	30	0	3.77
40.3	27.5	22.5	60	1	3.87
41.4	27.5	27.5	90	0	4.34
42.3	27.5	32.5	60	1	4.53
43.2	32.5	2.5	30	2	3.83
43.3	32.5	2.5	60	1	3.46
44.1	32.5	7.5	5	0	3.89
44.4	32.5	7.5	90	0	5.18
45.1	32.5	12.5	5	1	5.22
45.2	32.5	12.5	30	1	3.9
45.3	32.5	12.5	60	1	3.96
45.4	32.5	12.5	90	1	5.13
46.1	32.5	17.5	5	2	4.03
46.2	32.5	17.5	30	2	4.19
47.2	32.5	22.5	30	2	4.85
47.3	32.5	22.5	60	1	4.77
48.1	32.5	27.5	5	1	4.67
49.4	32.5	32.5	90	1	4.48

Figure S.1
Violin plots of the the log(CFU) for each sampling round and sampling depth. The white dot indicates the median of the data; the back rectangular box indicates the interquartile range; the thin horizontal line is the 95% confidence interval; the grey area is a kernel density distribution, i.e. an estimation of the probability density function of log(CFU).

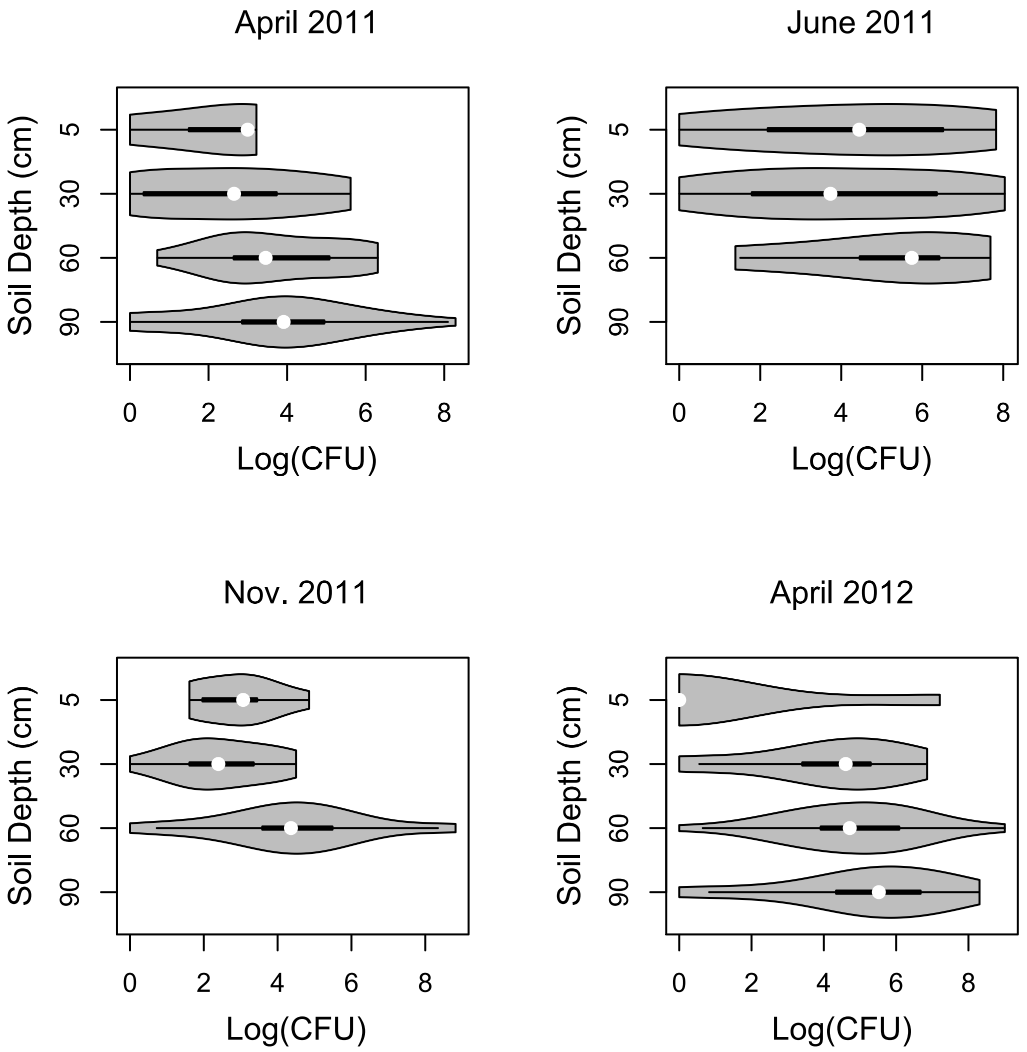

Figure S.2

Directional semivariograms for log-transformed counts (cfu g^{-1} soil + 1) of *B. pseudomallei* as a function of lag distance (in meters). (a) April 2011 (b) June 2011 (c) November 2011 (d) April 2012. Dots and circles correspond to E-W and N-S direction, respectively; thick and thin dashed lines represent the ordinary least square models fitted to the E-W and N-S directional semivariograms, respectively. Range of spatial autocorrelation for log CFU count was 7.6 meters, and range of spatial autocorrelation for presence of the organism was 7.1 meters.

a

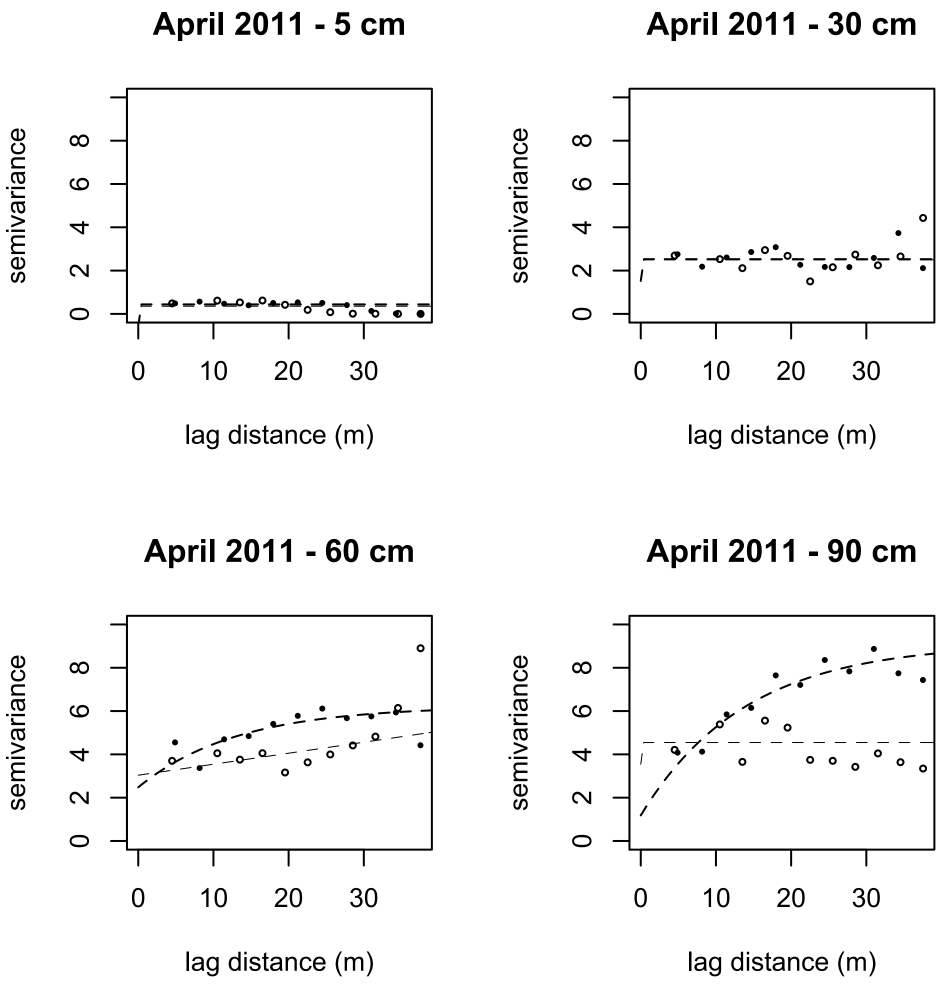

b

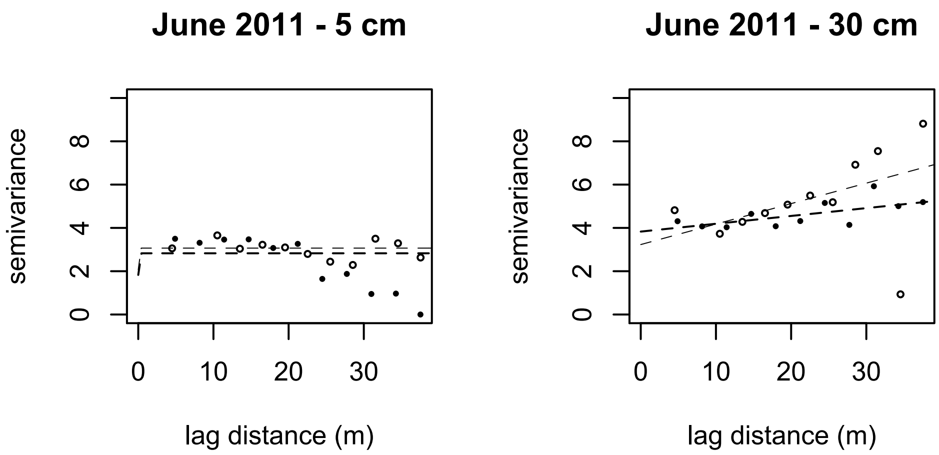
June 2011 - 5 cm
June 2011 - 30 cm
semivariance
0
2
4
6
8
0
10
20
30
lag distance (m)

c

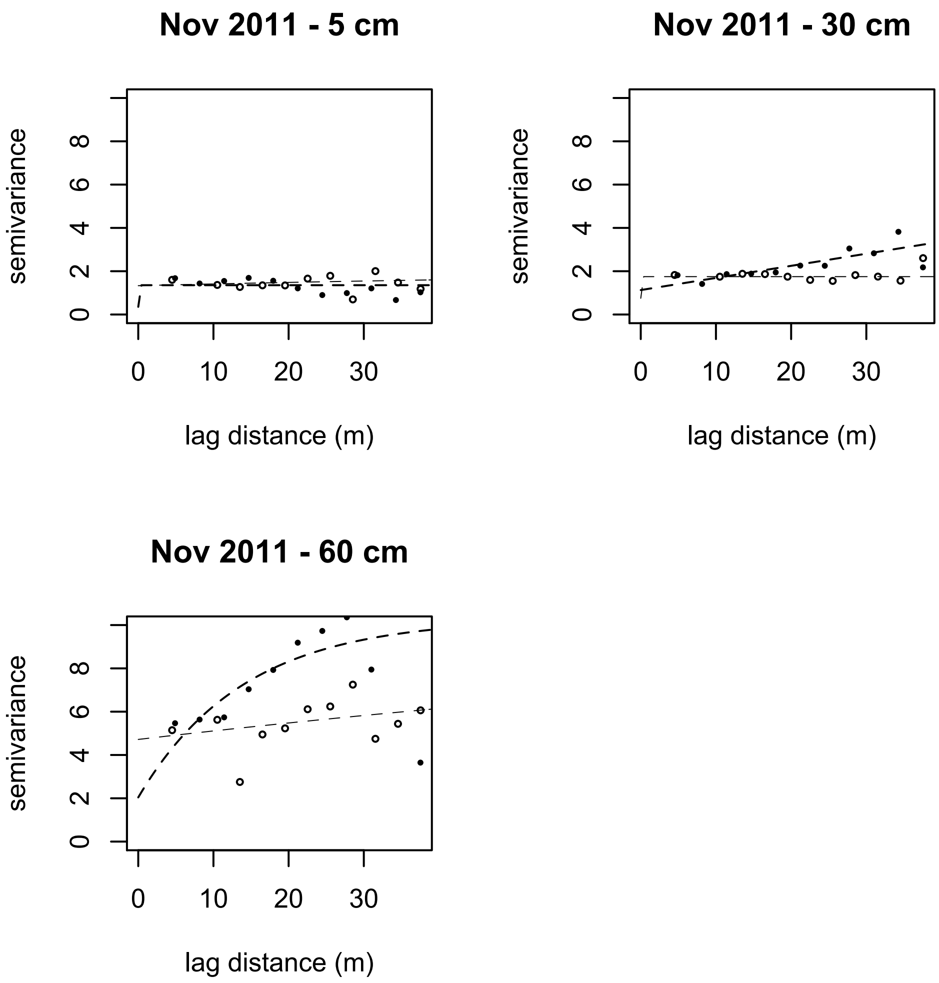
Nov 2011 - 5 cm
Nov 2011 - 30 cm
Nov 2011 - 60 cm
semivariance
lag distance (m)
0
2
4
6
8
10
20
30

d

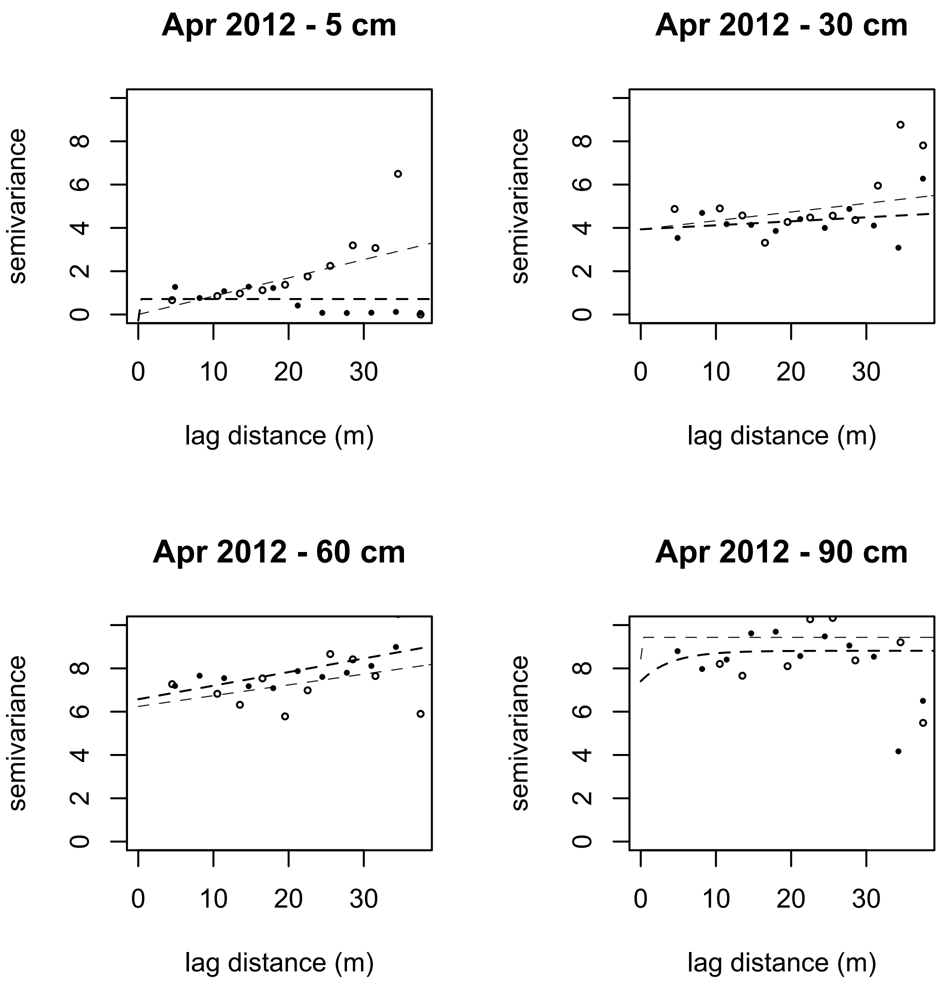

Apr 2012 - 5 cm
Apr 2012 - 30 cm
Apr 2012 - 60 cm
Apr 2012 - 90 cm
semivariance
lag distance (m)
0
10
20
30
0
2
4
6
8
